# A novel NIR-image segmentation method for the precise estimation of above-ground biomass in rice crops

**DOI:** 10.1371/journal.pone.0239591

**Published:** 2020-10-05

**Authors:** Julian D. Colorado, Francisco Calderon, Diego Mendez, Eliel Petro, Juan P. Rojas, Edgar S. Correa, Ivan F. Mondragon, Maria Camila Rebolledo, Andres Jaramillo-Botero

**Affiliations:** 1 School of Engineering, Pontificia Universidad Javeriana Bogota, Bogota, Colombia; 2 The International Center for Tropical Agriculture -CIAT, Palmira, Colombia; 3 INRAE-AFEF, I2S, LIRMM-ICAR, Université de Montpellier, Montpellier, France; 4 CIRAD, AGAP-Pam, Montpellier, France; 5 Chemistry and Chemical Engineering Division, California Institute of Technology, Pasadena, CA, United States of America; 6 Electronics Engineering and Computer Science Department, Pontificia Universidad Javeriana Cali, Bogota, Colombia; University of Wisconsin Madison, UNITED STATES

## Abstract

Traditional methods to measure spatio-temporal variations in biomass rely on a labor-intensive destructive sampling of the crop. In this paper, we present a high-throughput phenotyping approach for the estimation of Above-Ground Biomass Dynamics (AGBD) using an unmanned aerial system. Multispectral imagery was acquired and processed by using the proposed segmentation method called GFKuts, that optimally labels the plot canopy based on a Gaussian mixture model, a Montecarlo based K-means, and a guided image filtering. Accurate plot segmentation results enabled the extraction of several canopy features associated with biomass yield. Machine learning algorithms were trained to estimate the AGBD according to the growth stages of the crop and the physiological response of two rice genotypes under lowland and upland production systems. Results report AGBD estimation correlations with an average of *r* = 0.95 and *R*^2^ = 0.91 according to the experimental data. We compared our segmentation method against a traditional technique based on clustering. A comprehensive improvement of 13% in the biomass correlation was obtained thanks to the segmentation method proposed herein.

## 1 Introduction

Accurate and precise high-throughput phenotyping platforms are necessary to enable high-resolution linkage mapping for training genomic selection models in plant improvement [1, 2]. In rice, several morphological and physiological characteristics require spatio-temporal precise measurement for that purpose. Biomass is a key variable for quantifying grain yield and assessing crop health status. To overcome the limitations of traditional destructive methods for biomass sampling, above-ground methods to capture several canopy traits have gained traction. Most of the existing body of work uses near-infrared (NIR) aerial images for the calculation of canopy light reflectances at different wavelengths [3–5]. In this regard, a diverse set of Vegetation Indices (VIs) that highly correlate with the Above-Ground Biomass Dynamics (AGBD) have emerged. The use of high-quality multispectral aerial imagery has enabled the estimation of the AGBD by using Unmanned Aerial Vehicles (UAVs) [6–9].

In [10], a lightweight UAV was used for the above-ground estimation of biomass and panicles of rice. Spatio-temporal variations in several VIs were analyzed by fusing both visual (RGB) and multispectral (NIR) images into a single crop surface model. Linear regressions models were used for correlating the VI variations with the Above-Ground Biomass (AGB). Experimental results determined that both MVARI and VDVI indices enabled higher AGB estimations (*R*^2^ = 0.9), mostly due to the computation of the crop surface model with fused VIS/NIR imagery. However, the approach in [10] required expensive offline image processing calculations.

Several authors have also tackled the estimation of plant growth-related traits by data fusion from different sensors [11–14] for the computation of crop surface models based on image mosaicing methods [15–18]. Other approaches rely on the computation of individual aerial images. In this regard, several techniques for plot segmentation and image registration have enabled real-time image processing for the extraction of relevant features associated with the leaf/canopy biomass. In general, traditional methods based on edge detection thresholding, color histograms and clustering (otsu, K-means, watershed) are used in agriculture for plot segmentation [19–21]. Recently, the advent of low-cost UAVs with powerful computing capabilities has enabled more precise and sophisticated methods for image segmentation. In [22], a semantic segmentation method based on fully convolutional networks was proposed to extract features from RGB images that enable the classification of pixels corresponding to rice leaves, background, and weeds in the paddy field crops. The method achieved an average accuracy of 92%. Others have used machine learning methods for plot segmentation, and classification [23, 24]. All of these methods require training stages that limit real-time functionality.

The combination of several VIs to associate the biomass and grain yield with the light reflectance variations captured at different wavelengths. In [25], rice grain yield was predicted based on the dynamic changes in VIs directly calculated from a spectrometer device. Both linear and sigmoid-style dynamic models were found relating the spectral indices with the grain yield, demonstrating the accuracy of the selected VIs (*R*^2^ > 0.9 and *RMSE* < 5%). The proposed mechanisms in [26, 27] have also applied similar mathematical models for fitting linear relationships between several combined VIs and the physiological crop variables. Other authors have reported the use of machine learning methods to predict crop yield based on the non-linear relations obtained with narrow-band vegetation indices [28], as well as above-ground biomass estimation using classical vegetation indices [7, 8].

Our preliminary work in [29] presented a comprehensive survey from the specialized literature to identify which VIs were suitable for estimating rice biomass as a function of the growth stage of the crop. Seven spectral VIs were calibrated and combined in the form of multi-variable linear regressions for the estimation of the AGBD. Three different mathematical regressions were determined for each crop stage independently. Also, the K-means clustering classification was used for plot segmentation. Experimental results reported an average AGBD correlation of 0.76 compared with the biomass measurements taken with the traditional manual destructive method (ground-truth data).

**Fig 1:**
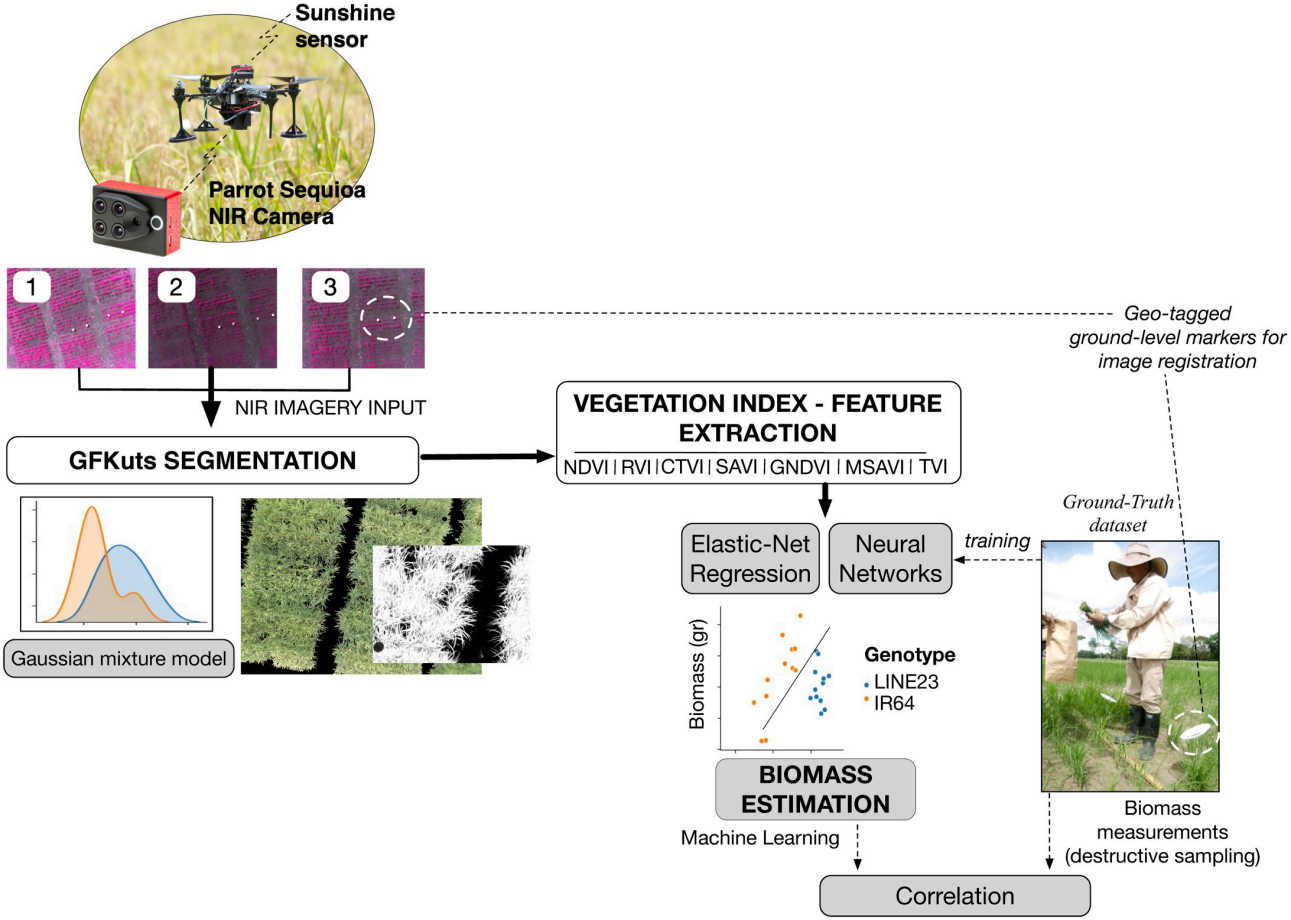
UAV-driven remote sensing of above-ground biomass in rice crops based on NIR imagery.

In this work, we present a comprehensive architecture for the estimation of the AGB in rice crops, as detailed by Fig 1. Multispectral imagery is captured and segmented by using a novel NIR-image segmentation method called GFKuts. This method solves an optimization problem using an energy function that allows the proper labeling of texture in the NIR image by using a Gaussian mixture model. After applying GFKuts, we use a second refinement process based on a Guided-Filter by taking into account information from all band channels of the multispectral camera: green, red, red-edge and near-infrared. The resultant NIR image-mask includes only relevant pixel information that accurately represents the canopy for the estimation of the AGBD. In turn, several VIs formulas are calculated and used as features for training our machine learning algorithms. Elastic-Net regressions are used to identify the canopy biomass according to the physiological response of two rice genotypes: Line23 and IR64 varieties, whereas neural networks are trained to estimate the AGBD according to the growth stages of the crop: vegetative, reproductive and ripening. Here, we addressed two challenges:

- The introduction of the GFKuts algorithm for the precise segmentation of NIR imagery with richer detail of the canopy structure, enabling improvements in the estimations of the AGBD.
- The integration of elastic-net regressions and neural networks to process nonlinear biomass dynamics with the calculations of the VIs during all stages of crop growth, and the association of physiological responses for two rice genotypes: Indica (IR64) and the Tropical Japonica subspecies (Line23).

## 2 Materials and methods

### Rice crops and UAV System

**Fig 2:**
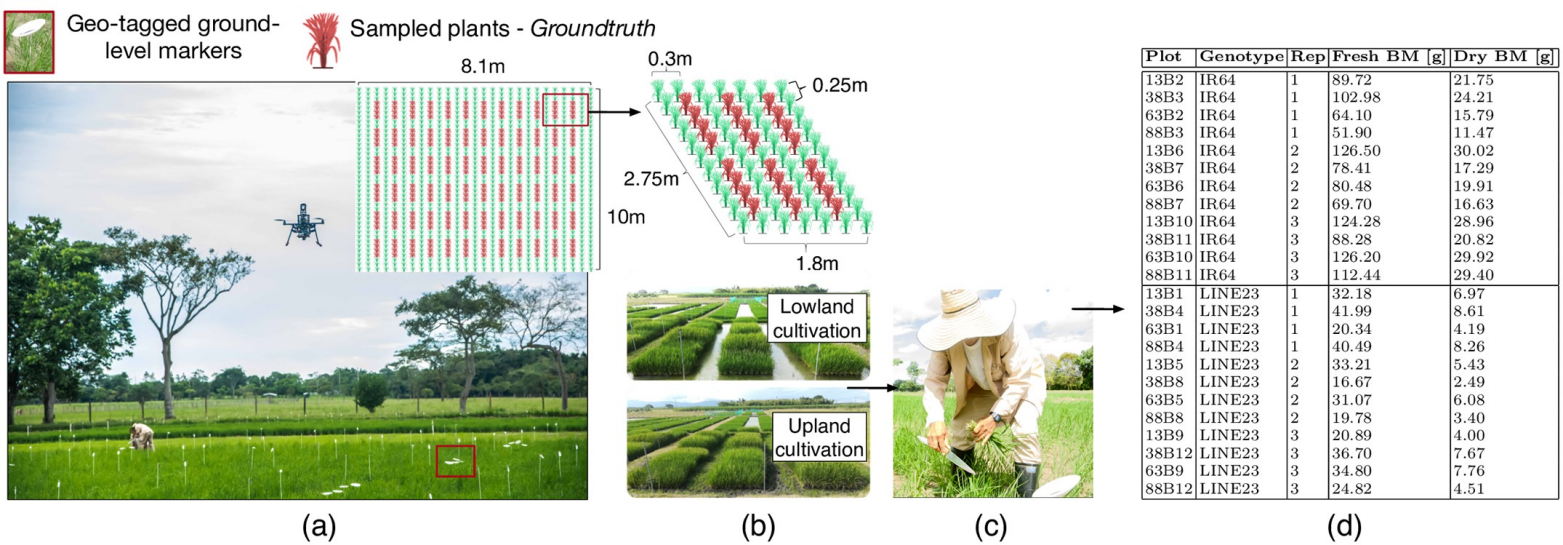
Crop setup: (a) Rice crops. (b) Each plot was designed with an area of 4.95*m*^2^. (c) Destructive biomass sampling. (d) An example of a Ground-Truth biomass (BM) dataset. The crop field was designed with three spatial repetitions (Rep) containing 2 contrasting rice genotypes.

Fig 2(a) describes the rice crop setup. The crops were designed with 3 spatial repetitions containing two rice varieties (genotypes) contrasting to biomass accumulation and flowering cycle: genotypes Indica (IR64) and the Tropical Japonica subspecies (Line23). IR64 is adapted to flooded rice paddies (lowland cultivation) whereas Line23 to dry soils (upland cultivation).

As shown in Fig 2(b), each plot was designed with a distance between plants of 25*cm* and 30*cm* between rows. Within each plot, we defined 6 linear sampled areas conformed by four plants, where ground-level markers were located and geo-referenced to enable air-ground image registration. Both rice varieties were combined within the same plots. Furthermore, both lowland and upland rice production systems were designed and implemented to assess the biomass dynamics during the entire phenological cycle of the crop, ranging between 95–110 days. This cycle was divided into three growing stages: vegetative, reproductive, and ripening.

Here, we report on 72 sampled areas, evaluated from the vegetative through the ripening stage, capturing around 2.000 NIR images per crop stage, yielding a dataset of 6.000 images per trial. We conducted two trials (3 months each) of in-field testing, in which our UAV platform performed 10 flights per crop stage, capturing an overall of 12.000 images. In this regard, the machine learning algorithms applied for the estimation of biomass (as detailed in Fig 1) used 60% of the dataset for training, whereas the testing accounted for the remaining 40% of the images.

Along with the UAV-based aerial sampling, we manually collected several plants corresponding to sampled areas previously mentioned. Fig 2(c) depicts this process for destructive biomass sampling. The Ground-Truth was defined by weighting the collected samples from each plot. The estimated dry weight of total aerial biomass was estimated after 3 days in the oven at 65°*C*. Fig 2(d) shows an example of the assembled Ground-Truth database.

**Fig 3:**
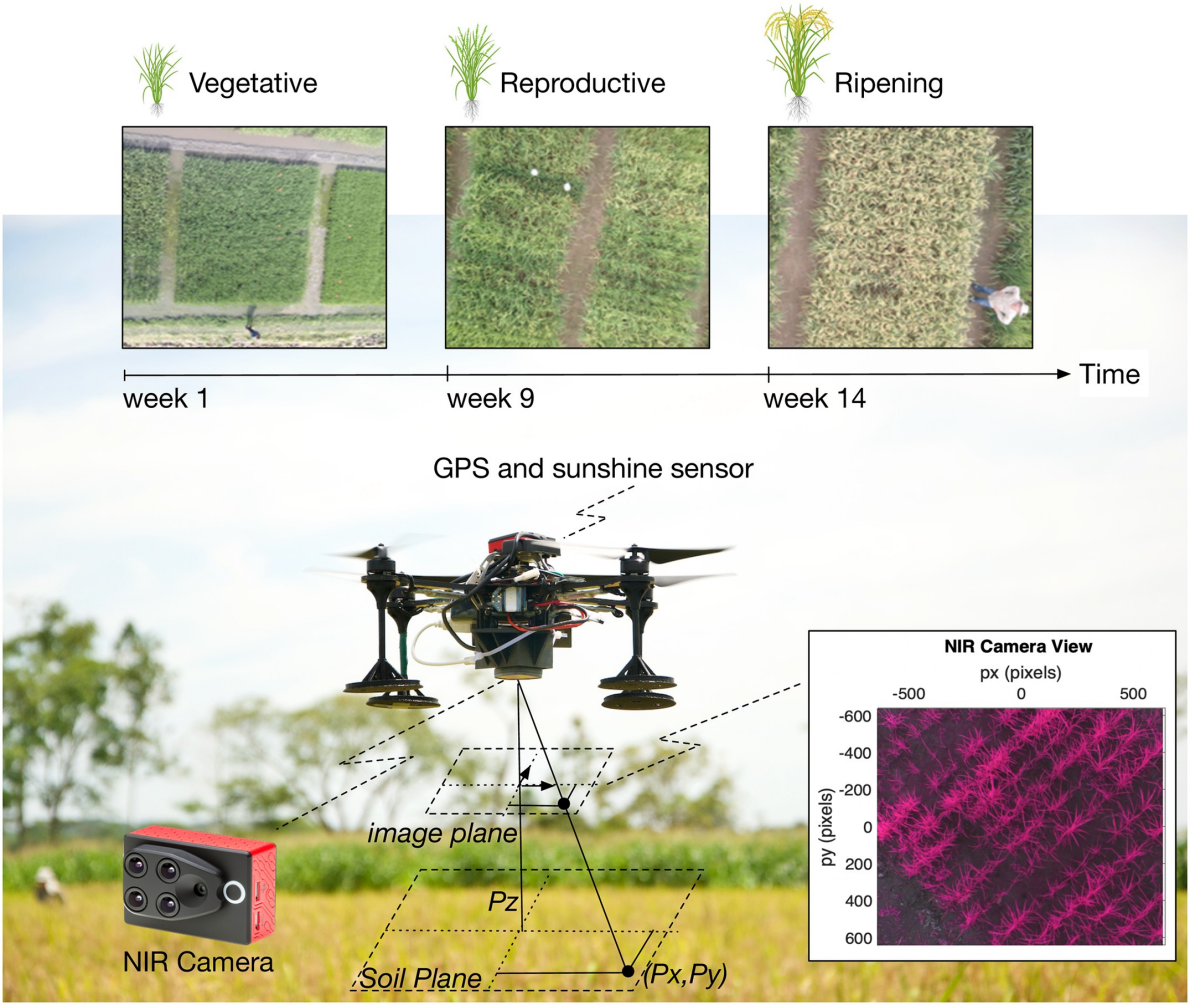
Detailed presentation of the UAV system utilized in this work.

Our aerial samples were acquired with the commercial quadcopter UAV Asctec Hummingbird, manufactured by INTEL’s Ascending Technologies GmbH. By using the UAV’s onboard ARM High Level Processor (HLP), we integrated the Parrot Sequoia multispectral sensor. Fig 3 gives a detailed presentation of the UAV setup.

Next, we present how the proposed GFKuts segmentation technique enables the precise selection of the crop areas under study, during each crop stage. The GFKuts method solves an optimization problem for properly labeling texture and color information using a Gaussian mixture model. It relies on a Guided-Filter refinement process that requires both NIR and RGB imagery. The Parrot Sequoia camera delivers high resolution RGB images with 4608 × 3456 pixels enhancing the resultant NIR image-mask, since the other 4 spectral sensors of the camera deliver images with 1280 × 960 pixels in resolution.

### GFKuts-driven image segmentation

This section introduces our proposed algorithm, GFKuts, which consists of: (i) a modified version of the GrabCut algorithm [30], fed with a pair of binary masks obtained from a Montecarlo-sampled K-means segmentation over the image [31], (ii) followed by a refinement step using Guided Filtering [32] to smooth the pixel information associated with the plant’s canopy. The proposed algorithm can operate in the sRGB color, with single-channel images, or a custom composed-channel images according to a desired VI to be estimated, e.g., concatenating a 3-channel image with multispectral images.

Segmentation algorithms can be divided in two types, hard and soft, according to the output mask. Hard segmentation algorithms create a binary output with only two levels for the background and foreground, while soft segmentation algorithms create a set of levels between those two. In GrabCut, the segmentation is represented in each pixel by a set of levels $\underline{\alpha }=\left(\alpha_{1},\ldots ,\alpha_{N}\right)$. For GrabCut, as a hard segmentation algorithm, $\underline{\alpha }$ takes only two values {1, 0}.

#### GrabCut algorithm

The GrabCut image segmentation method, was proposed as a semi-manual and iterative method to improve the segmentation in each iteration of the binary mask. GrabCut uses an interactive foreground extraction methods to improve over the original GraphCut method [33]; changing the energy minimization method and the initialization steps. Grabcut requires the creation of three image-masks: one binary mask for the background (*T*_*B*_), one binary mask for the foreground (*T*_*F*_) and a final mask with uncertainty pixels (*T*_*U*_), that can be binary or have more quantization levels. These three mask are known as the trimap.

GrabCut uses an internal representation based on a Gaussian Mixture Model (GMM) to store image probability of a pixel being segmented. This model allows for a three-color channel, as well as, monochrome input images. The energy minimization is applied several times in an iterative process to improve the results. As most global optimization methods in computer vision [34], GrabCut uses an energy framework that minimizes a function using two parts: (i) a data function *U*() and (ii) a smoothness function *E*(). The minimum cut algorithm is used by both GraphCut and GrabCut methods in order to obtain the hard segmentation that finds for an $\underline{\alpha }$ that minimizes Eq 1.

$$\begin{array}{c} E\left(\underline{\alpha },k,\underline{\theta },z\right)=U\left(\underline{\alpha },k,\underline{\theta },z\right)+V\left(\underline{\alpha },z\right). \end{array}$$ (1)

The first function *U*() is called the data function, which measures the fit between the segmentation and the image values **z**. The terms { $\underline{\theta }$ and **k**} correspond to the GMM parameters. The smoothness function *E*() evaluates the dissimilarity of neighbouring pixels and depends only on the hard segmentation $\underline{\alpha }$ and the image values **z**. Further details on the method described by Eq 1 can be found in [34].

The original Grabcut paper and its implementation follows a procedure that involves 3 steps:

1. An initialization of the trimap by manually supplying a rectangular region of *T*_*B*_. The foreground *T*_*F*_ is set to zero, $T_{U}=\bar{T_{B}}$ the values of *α* are initialized with the supplied *T*_*B*_.
2. Min-cut optimization over the model.
3. Ask the user for new points for *T*_*B*_ and *T*_*F*_ to refine the segmentation, and repeat the optimization until the user approves the convergence.

GrabCut is widely used for its ease of implementation and for the excellent results in generating a binary classification, however, it suffers from the drawback of being a semi-manual algorithm. Like other global optimization methods, the main advantage of a GMM model and the min-cut optimization relies on the smooth segmentation of the image and the fast-growing convergence. By applying the Montecarlo Sampled K-means, we provide an initial mask to GrabCut that does not require any user input.

**Algorithm 1** Montecarlo Sampled K-means.

the input of the algorithm is the image **z** and the number of samples *l*

**for** Each pixel in range (1 … *l*) **do**

 Select a random pixel from **z**

 Append its value to **z**_*l*_

 Store its coordinates in **z**_*x*,*y*_

**end for**

Run a binary K-means over **z**_*l*_ to get the labels **z**_*l*,0_ and **z**_*l*,1_

**if** length of (**z**_*l*,0_) > length of (**z**_*l*,1_) **then**

 Create a mask *T*_*F*_ and set the coordinates in **z**_*x*,*y*_ of each pixel in **z**_*l*,0_ as the foreground (in our case, the canopy).

 Create a mask *T*_*B*_ and set the coordinates in **z**_*x*,*y*_ of each pixel in **z**_*l*,1_ as the background (in our case, the soil).

**else**

 Create a mask *T*_*F*_ and set the coordinates in **z**_*x*,*y*_ of each pixel in **z**_*l*,1_ as the foreground (in our case, the canopy).

 Create a mask *T*_*B*_ and set the coordinates in **z**_*x*,*y*_ of each pixel in **z**_*l*,2_ as the background (in our case, the soil).

**end if**

#### Montecarlo sampled K-means

The Montecarlo methods are a broad class of computational algorithms that rely on random sampling until a particular fitness function is met.

K-means on the other hand is a clustering algorithm, usually integrated in image segmentation techniques to separate colors. The main drawback of using K-means for binary segmentation is the lack of spatial coherence in the result. K-means can be classified as a local algorithm and can be heavily affected by under or overexposed regions, shadows or noise.

The proposed Montecarlo Sampled K-means works as follows: a subset of pixels denoted as **z**_*l*_ are randomly selected by the algorithm in order to separate the image values *z* into two groups, where the subscript *l* indicated the length of the selected array. The clustering is based on the colors associated with each pixel.

These two new clusters follow all the desired properties for a (*T*_*B*_,*T*_*F*_) initialization trimap. In our context, plot images have two important characteristics: (i) large areas correspond to the canopy, and (ii) minor areas correspond to soil and exogenous elements. The largest cluster will be related to the canopy, and the other to soil areas. Each sampled pixel **z**_*l*_ is associated with its respective position *z*_*x*,*y*_ in order to generate masks that will be used later as *T*_*B*_ and *T*_*F*_.

**Fig 4:**
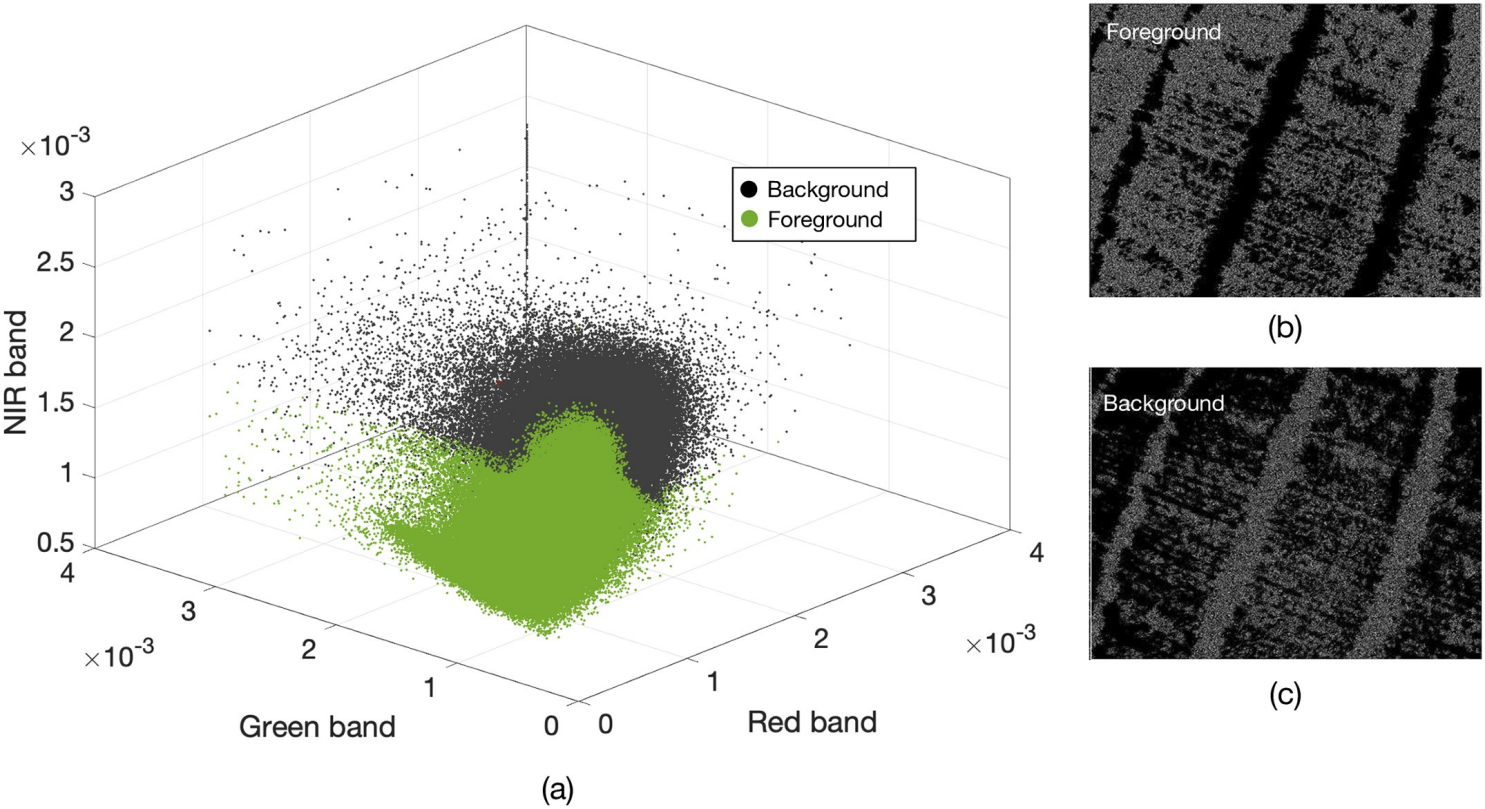
The two clusters in the RGN space, the foreground (*T*_*F*_) and background (*T*_*B*_), created following Algorithm 1. The segmented RGN image was captured using the Parrot-Sequoia, stacking the respective multi-spectral camera bands, after aligning the images according to the camera intrinsics and drone altitude.

**Fig 5:**
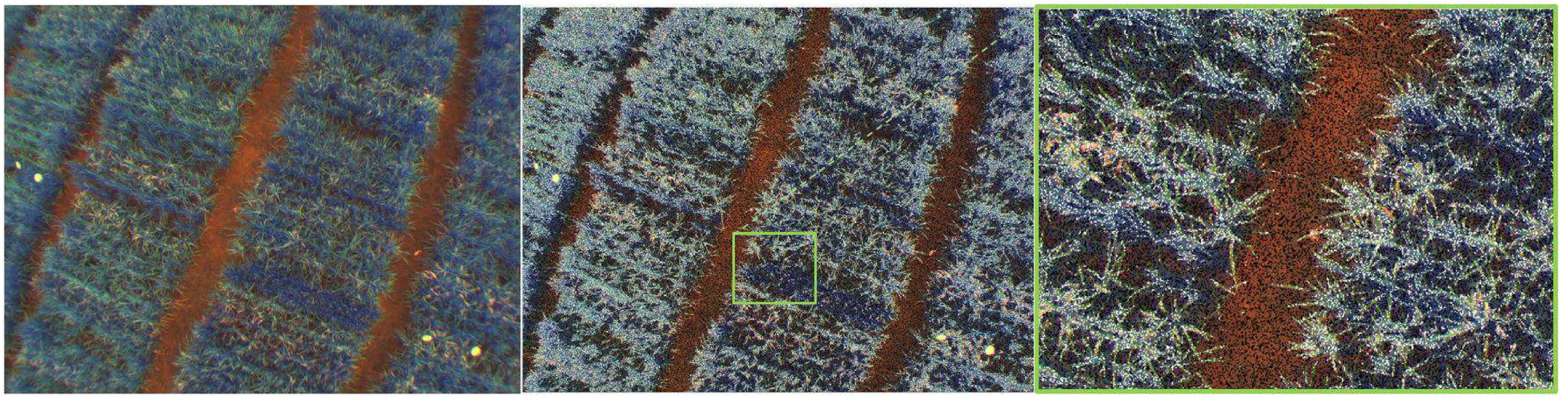
Uniform random distribution of grouped and classified pixels on RGN image. White pixels are associated with vegetation, while black pixels are associated with soil.

As seen in Algorithm 1, a random selection of pixels in the image is made using their spatial dimension. Applying a binary K-means algorithm to this set of pixels, will group a scattered distribution of samples uniformly over the image. The above process can be applied to images with one or three channels. Fig 4 shows an example of these two clusters in the Red-Green-NIR (RGN) color space obtained with K-means and the *T*_*B*_ and *T*_*F*_ masks. Fig 5 presents an image in the RGN color space as a reference, in which the samples follow a uniform distribution.

#### Guided filter refinement

**Fig 6:**
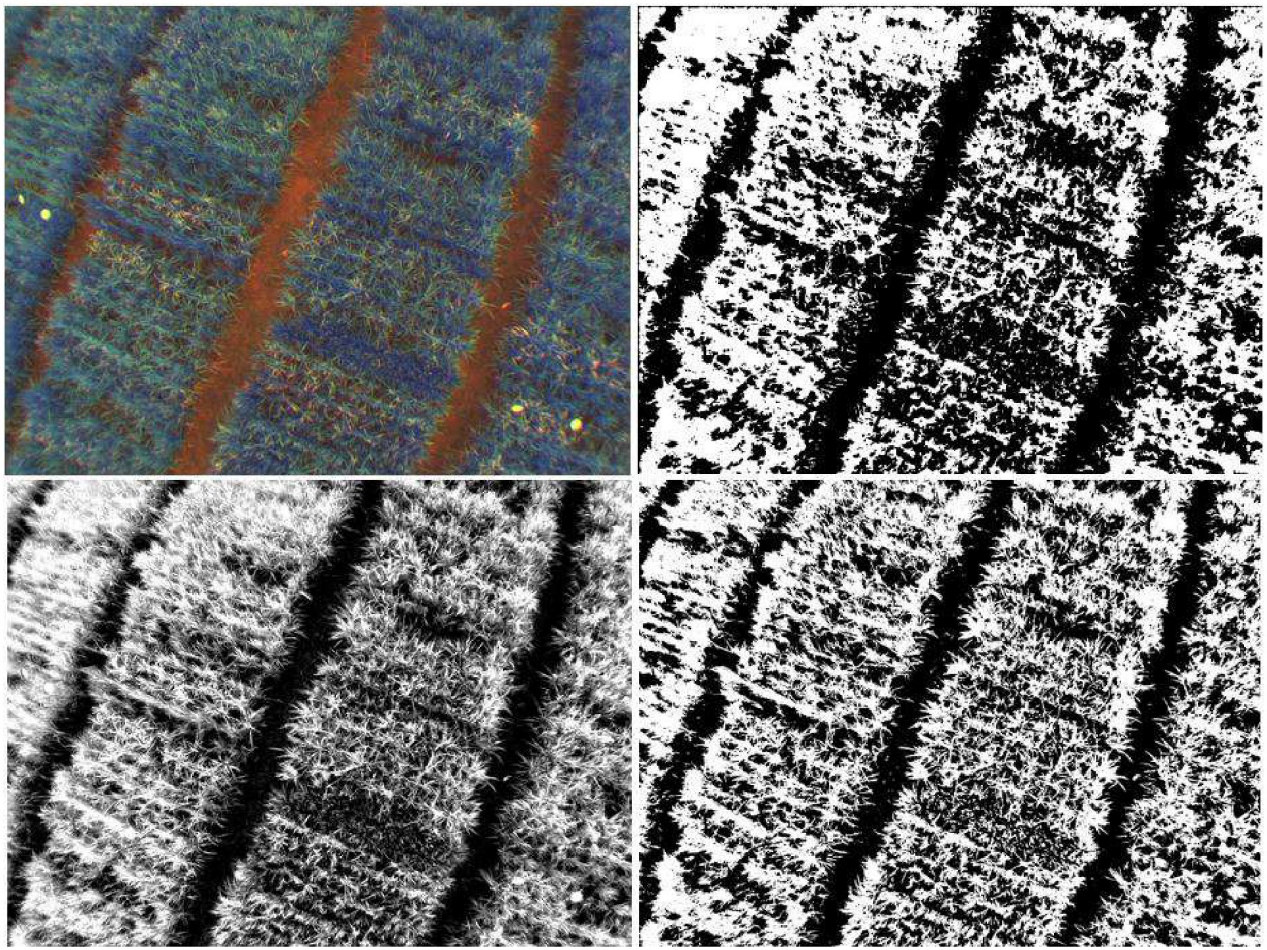
(a-topleft) The original image in RGN color space, (b-topright) the hard segmentation output of the GFkuts algorithm after 5 iterations, (c-bottomleft) the soft segmentation result of the GF refinement, (d-bottomright) and the adaptive thresholding output to create a binary mask of the canopy.

Given the binary output of GrabCut and the complex structure of the canopy in which the leaves create an elaborate net of high-frequency formations (see Fig 6), it is necessary to refine the masks to follow the structures created by the canopy. With this in mind, the Guided Filtering emerges as an alternative that involves a fast and local method, with a performance similar to techniques based on global optimization such as anisotropic diffusion [35], yet with an *O*(*n*) computational complexity.

The Guided Filter (GF) [32] is a non-linear filter in which the convolutional kernel changes according to the spatial and radiance characteristics of the image. Similarly to others convolutional filters, the GF output *q* (for each image pixel *p*), can be expressed as a weighted average across the convolutional window *W*_*ij*_, as shown in Eq 2, where *i*, *j* designates the pixel coordinates of the input and output images.

$$\begin{array}{c} q_{i}=\sum_{j}W_{ij}p_{j} \end{array}$$ (2)

The existence of a variable window implies that it may depend on a second input image, this is known as the guidance image. We exploited this approach to filter the output of the GrabCut algorithm and refine its binary segmentation with the original image as reference. The weight used by the GF is determined by using Eq 3.

$$\begin{array}{c} W_{ij}^{GF}\left(I\right)=\frac{1}{{\left|\omega \right|}^{2}}\sum_{k;\left(i,j\right)\in \omega_{k}}(1+\frac{\left(I_{i}-\mu_{k}\right)\left(I_{j}-\mu_{k}\right)}{\sigma_{k}^{2}+\epsilon }) \end{array}$$ (3)

The term $W_{ij}^{GF}$ depends on the reference image *I*. The parameters *μ*_*k*_ and $\sigma_{k}^{2}$ are the guidance-image mean and variance estimated over a window *w*_*k*_, *ϵ* is called a regularization parameter and |*ω*| counts the number of pixels in the window *w*_*k*_. However, the direct calculation of the window $W_{ij}^{GF}$ is not performed due to its high computational cost. The implementation of the GF follows an *O*(*n*) procedure detailed by Algorithm 2.

**Algorithm 2** Guided Filter Calculation.

*E*_*r*_(*I*) denotes a function that calculates the image mean over a radius *r*, *ϵ* is a regularization parameter, the operations .* and ./ denotes the matrix element-wise calculation, and *q* is the image output.

 **Step 1**: Input image *p*, input guidance *I*, radius *r* and regularization *ϵ*.

 1: *μ*_*I*_ ← *E*_*r*_(*I*), *μ*_*p*_ ← *E*_*r*_(*I*), *Corr*_*I*_ ← *E*_*r*_(*I*. * *I*), *Corr*_*Ip*_ ← *E*_*r*_(*I*. * *p*).

 2: $\sigma_{I}^{2}\leftarrow Corr_{I}-\mu_{I}$. * *μ*_*I*_, $\sigma_{Ip}^{2}\leftarrow Corr_{Ip}-\mu_{I}$. * *μ*_*p*_

 3: $a\leftarrow \sigma_{Ip}^{2}./\left(\sigma_{I}^{2}+\epsilon \right)$, *b* ← *μ*_*p*_−*a*. * *μ*_*I*_

 4: *μ*_*a*_ ← *E*_*r*_(*a*), *μ*_*b*_ ← *E*_*r*_(*b*)

 5: *q* = *μ*_*a*_. * *I* + *μ*_*b*_

#### GFKuts algorithm

The proposed algorithm, GFKuts, integrates the methods previously explained: (i) the Montecarlo Sampled K-means, (ii) the optimization and modeling of GrabCut, (iii) the GF refinement, and finally (iv) an adaptive threshold. GFKuts exploits the best characteristics of each one of these algorithms, by combining local and global methods, in order to obtain a detailed image of the canopy.

Fig 6 shows the results of each step of GFKuts. The sparse initialization of GrabCut creates a uniform surface on which the global optimization performed by min-cut can grow according to the canopy textures. In practice, at least five iterations are required to cover the entire image surface, as seen in Fig 6(b).

The GF refinement operates on the convolutional kernel vicinity and the guidance smooths the binary image according to its radiance and texture. This process is known as feathering and has the property of creating a grayscale mask or soft segmentation from a hard segmentation output. Finally, GFKuts performs an adaptive threshold of the soft segmentation output of the GF refinement, if a hard segmentation is needed. The entire GFKuts algorithm is detailed in Algorithm 3.

**Algorithm 3** GFKuts,

*z* is the plot input image, *l* is the number of samples used in K-means, *n* is the number of iterations of GrabCut, *r* is the GF radius, *ϵ* is the regularization.

 {*T*_*B*_, *T*_*F*_} ← MontecarloSampledK-means (*z*, *l*)

 **while** *α* converges or run *n* iterations **do**

  All pixels not set in *T*_*B*_ or *T*_*F*_ are set as a possible foreground pixels *T*_*UF*_

  *α* ← GrabCut(*z*, *T*_*B*_,*T*_*F*_)

  Use the segmented image *α* as the new possible foreground pixels *T*_*UF*_

 **end while**

 *α*_1_ ← GF (*Image* = *α*, *Guidance* = *z*)

 *α*_2_ ← adaptiveBinaryThreshold(*α*_1_)

## 3 Results and discussion

### NIR-image segmentation metrics

The performance of the proposed GFKuts method is evaluated by computing the Accuracy, Precision, Recall and F1-score. These metrics are also compared against traditional image segmentation methods such as Thresholding [41] and K-means [42], but also against the original GrabCut method [33].

**Fig 7:**
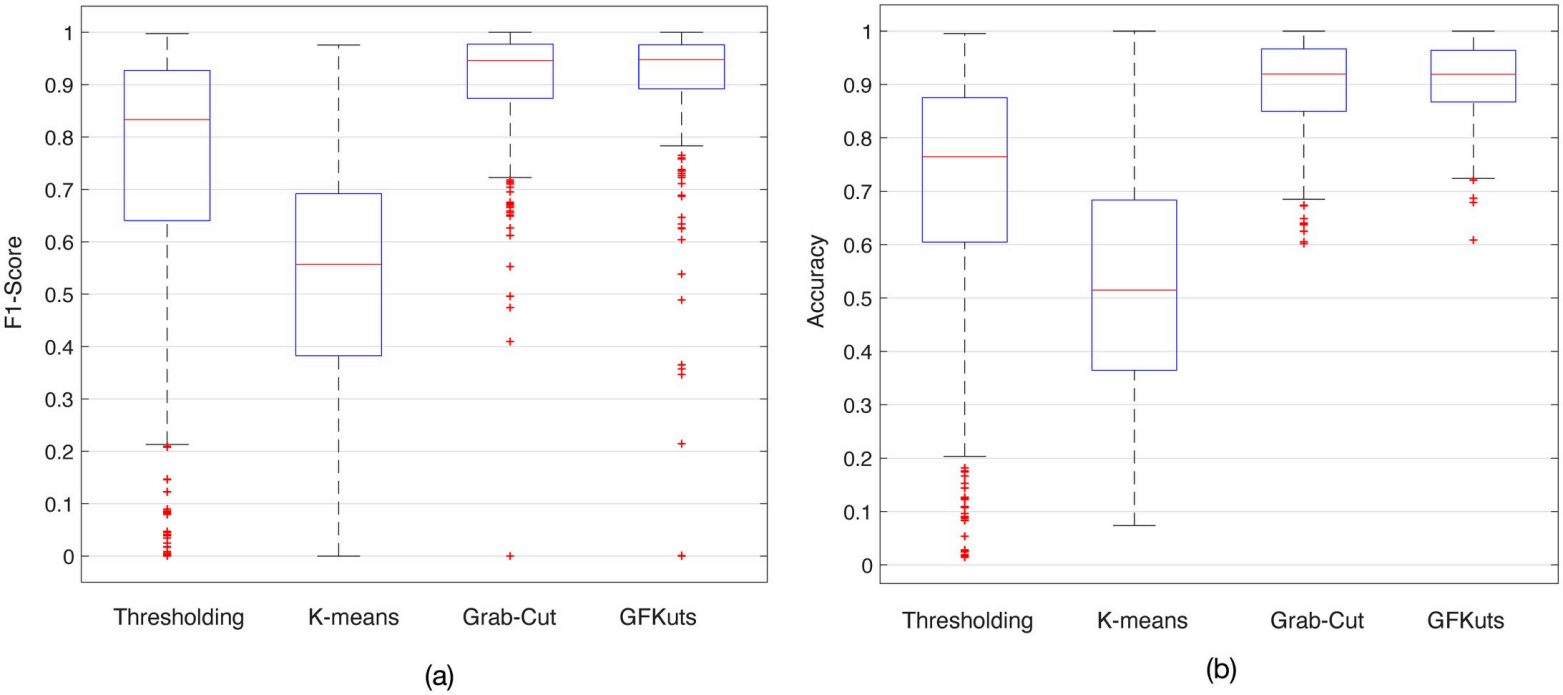
F1-score and accuracy for all tested algorithms reported in Table 1.

Table 1 contains the numerical results regarding the evaluation of the aforementioned segmentation algorithms. On average, the proposed GFKuts approach outperformed the other methods, concretely in comparison with the Thresholding and K-means. The overall performance data is also presented in Fig 7. As mentioned, the proposed GFKuts is based on the standard GrabCut method, that requires a manual input during the algorithm iteration in order to properly determine both background *T*_*B*_ and foreground *T*_*F*_ values (see Section 2). Given that, the original semi-manual GrabCut method also achieves higher performance in terms of F1-score and Accuracy.

**Table 1:** Image segmentation performance. Mean results over 400 NIR images (image sub-regions of 10 × 10 pixels).

	Thresholding	K-means	GrabCut	GFKuts
Accuracy	0.701	0.54	0.898	0.91
Precision	0.845	0.95	0.951	0.954
Recall	0.729	0.457	0.92	0.93
F1-score	0.778	0.605	0.936	0.942

**Fig 8:**
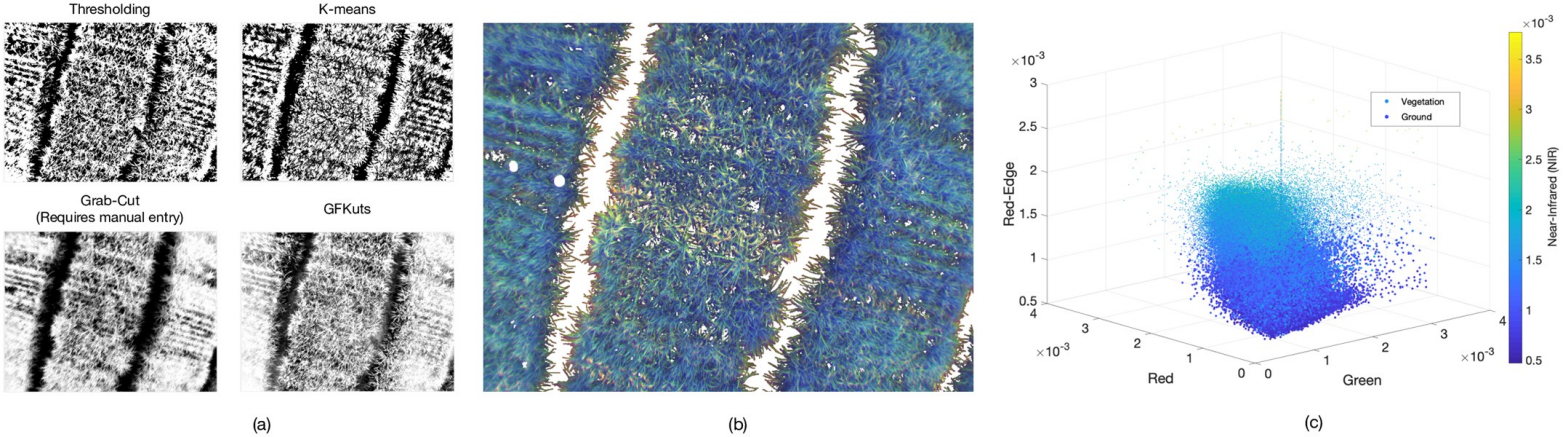
Rice canopy detail after the segmentation process: (a) segmentation results for each tested algorithm. (b) reconstructed image using four channel data space depicted in (c) RGN+(red-edge) space.

On the other hand, our GFkuts method is fully automatic, using the Montecarlo sampled K-means described in Algorithm 1 to optimally separate the vegetation from the soil, as shown in Fig 4. As detailed in Fig 7(a), GFkuts slightly improved over GrabCut in terms of data dispersion and F1-score, i.e., the mean between precision and recall. Thanks to the GFKuts segmentation presented herein, our system counts with an optimal, accurate and automatic method for NIR imagery segmentation, enabling richer detail of the rice canopy that improves on the VI-based feature extraction and the estimation of above ground biomass, as detailed in Fig 8.

### Vegetative index

Vegetation Indices (VIs) are well-known parameters used to quantify several physico-chemical variables in plants, by associating spectral reflectances that are highly related to the variable of interest. Different wavelengths of light have a different level of plant absorption depending on the leaf composition given by several genetic traits. In particular, the relation between the VIs with the photosynthetic activity and canopy structural properties has allowed new methods for non-destructive Above-Ground Biomass Estimations (AGBE).

Table 2 presents the result of a comprehensive literature review, by selecting a set of VIs calculated from different wavelength reflectances, specially the green, red, red-edge and near infrared bands. The selected VIs exhibit a strong dependence on the NIR reflectance due to leaf chlorophyll absorption, providing a non-invasive approach to determine the health status of the plants and the canopy biomass. Most of the existing body of research focused on NIR-based above-ground biomass estimations [4, 10, 11], combine the information provided by several vegetation indices in order to avoid saturation issues. For instance the NDVI, which is one of the most common VIs used to assess the crop biomass, tends to saturate with dense vegetation. In turn, the NDVI alone is not accurate during the reproductive and ripening stages of rice growth. Here, by combining several VIs across the crop stages, we ensure to capture data on wavelengths located in the red-edge and another spectral reflectances that accurately express the healthy status of the leaves (higher NIR and green band readings).

**Table 2:** Near-infrared vegetation indices for non-destructive above-ground biomass estimations. The term *ρ*_*f*_ refers to the reflectance value at the frequency *f*).

VI	Formula
Normalized Difference Vegetation Index [36]	$\text{NDVI}=\frac{\rho_{780}-\rho_{670}}{\rho_{780}+\rho_{670}}$
Green Normalized Difference Vegetation Index [37]	$\text{GNDVI}=\frac{\rho_{780}-\rho_{500}}{\rho_{780}+\rho_{500}}$
Difference Vegetation Index [38]	DVI = *ρ*_780_ − *ρ*_670_
Corrected Transformed Vegetation Index [38]	$\text{CTVI}=\frac{\text{NDVI}+0.5}{\left|\text{NDVI}+0.5\right|}\sqrt{\left|\text{NDVI}+0.5\right|}$
Soil-Adjusted Vegetation Index [39, 40, 37]	$\text{SAVI}=\left(1+L\right)(\frac{\rho_{800}-\rho_{670}}{\rho_{800}+\rho_{670}+L})$ with L = 0.5
Modified SAVI [39, 40]	$\text{MSAVI}=\frac{1}{2}(2\rho_{800}+1-\sqrt{{\left(2\rho_{800}+1\right)}^{2}-8\left(\rho_{800}-\rho_{670}\right)})$
Simple Ratio [36]	$\text{SR}=\frac{\rho_{780}}{\rho_{670}}$

As previously mentioned, we used the spectral indices in Table 2 as the features to extract from the acquired aerial imagery. Since the estimation of the accumulated biomass depends on the accuracy and reliability of the extracted VI-based features, it is important to compare the correlations between the aerial and the ground-level features, i.e, canopy-plant scales. To this purpose, the VI-based features were calculated by applying the formulas in Table 2 to both ground-level and aerial samples. Given that, the former relies on the assembled ground-truth database described in Fig 2(d), while the latter relies on the canopy imagery.

**Fig 9:**
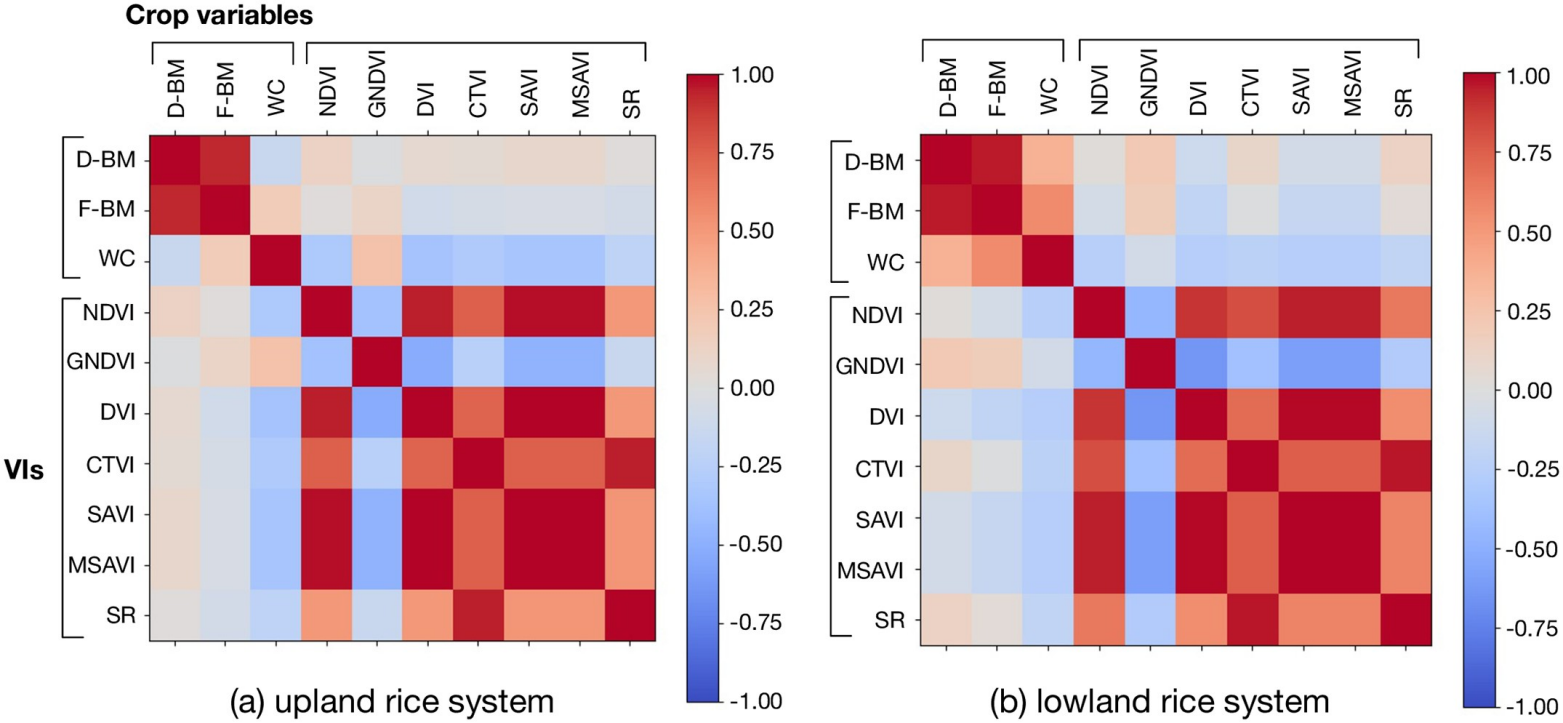
Correlation matrix for the extracted features for (a) up-land and (b) low-land production systems. The terms D-BM and F-BM correspond to the dry and fresh biomass, respectively. WC is the water content, while the rest of the features correspond to the Vegetation Indices (VIs) defined in Table 2.

Fig 9 presents the feature correlation results obtained for both rice production systems: upland (dry soils) and lowland (flooded paddies). Correlations were calculated using Eq 4, as follows:

$$\begin{array}{c} \begin{array}{c} r=\frac{\sum_{i=1}^{n}\left(x_{i}-\bar{x}\right)\left(y_{i}-\bar{y}\right)}{\sqrt{\sum_{i=1}^{n}{\left(x_{i}-\bar{x}\right)}^{2}{\left(y_{i}-\bar{y}\right)}^{2}}} \end{array} \end{array}$$ (4)

From Fig 9, two very interesting conclusions are found. First, some of the vegetation indices (VIs) calculated in an independent manner show positive correlations with dry- (D-BM) or fresh- (F-BM) biomass, as seen in the two first columns of the correlation matrices (specially with D-BM); to find a stronger correlation between VIs and biomass, the combination and calibration of several VIs is required. Given that, the dry biomass Ground-Truth measurements will be used in the training phase of our machine learning algorithms, along with the VIS calculations, to estimate dry biomass.

However, there is a strong correlation between different indices. For instance, MSAVI has a strong correlation (strong red squares) with NDVI, DVI and SAVI, in a similar way as CTVI is related to SR. The only VI that is clearly isolated is GNDVI. With this in mind, it would be useful to consider a dimensionality reduction in the number of inputs in order to reduce the computational complexity of these calculations. Considering a future on-board online implementation of our algorithms, such a scenario is extremely beneficial.

In order to analyze the variance of these features through an entire phenological cycle, we conducted several VI measurements. At canopy-level, several factors affect the spectral reflectances of the crop: solar radiation, plant morphology and color, leaf angles, undergrowth, soil characteristics and water. In our system, the Sequoia multispectral camera comes with an integrated sunshine sensor to compensate light variations in the resultant image. Also, the image segmentation method deals with the filtering of undergrowth and other soil noises. In this regard, the change in the leaves color is the most notably variation of the crop through the phenological cycle. Fig 3 highlights these changes. As previously mentioned, the maturation of the plants occur while the leaves begin to senesce, in the ripening stage.

**Fig 10:**
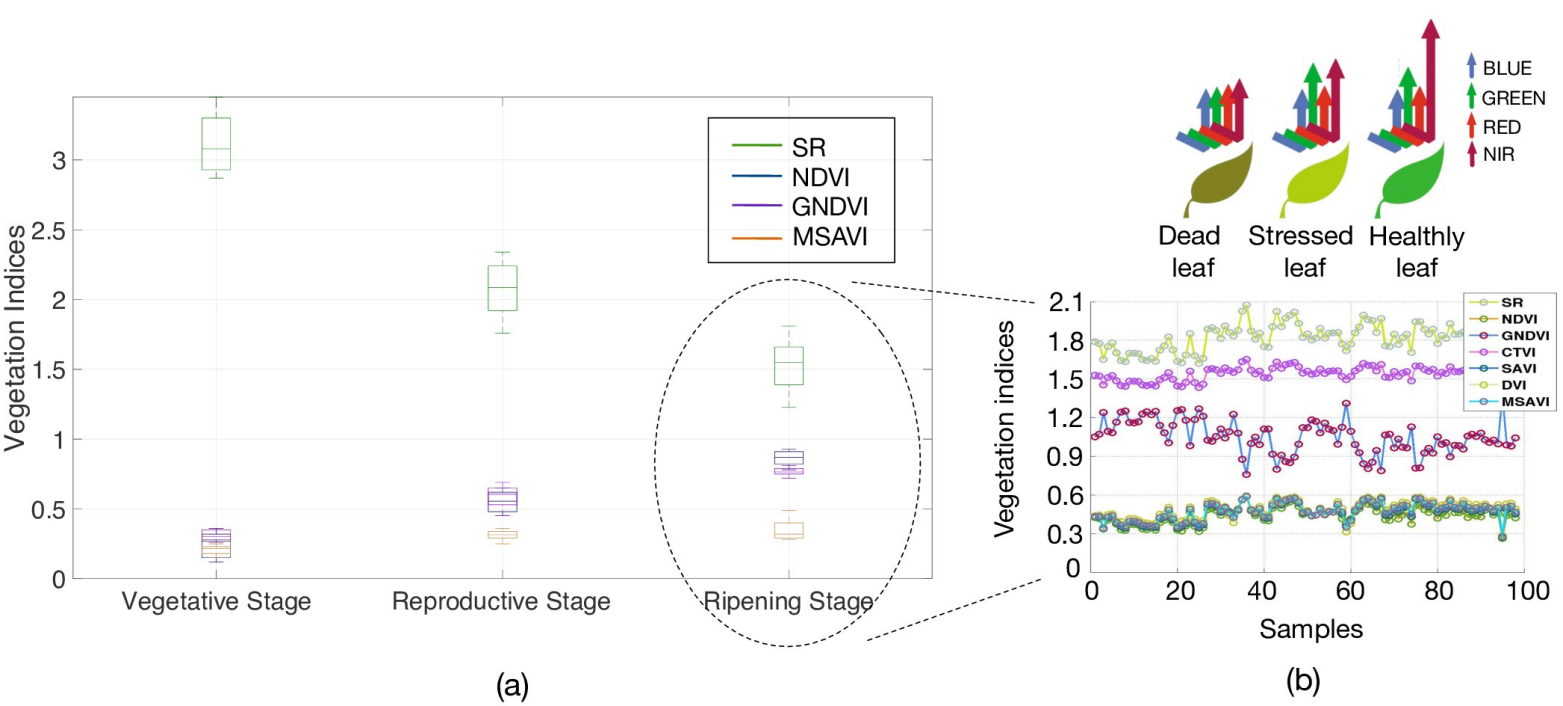
Vegetation Index computation: (a) VI variance through an entire phenological cycle. (b) An example of the VI-feature dynamics during a single growing stage. The inset shows the rice-leaf healthy status based on different wavelength readings.

Given that, it remains crucial to validate the accuracy of the selected VIs in terms of their variance during each crop stage. Fig 10 shows the results for our most representative VIs: SR, NDVI, GNDVI and MSAVI. Note from Fig 9 that these VIs exhibit some unique responses when comparing both crop production systems: upland or lowland. For instance, the GNDVI has an exclusive positive correlation with the biomass in both crop systems, being an unique feature since it has none correlation with other VIs. In general, the selected VIs shown in Fig 10(a) present a low variance through the entire phenological cycle, being reliable for our application. For this test, we computed the VIs from 360 random images per stage.

Next, we present the experimental results regarding the estimation of the AGBD using artificial neural networks trained with the selected VI-features. As previously mentioned in Section 2, datasets were divided 60% for training while the remaining 40% for testing.

### Biomass estimations

**Fig 11:**
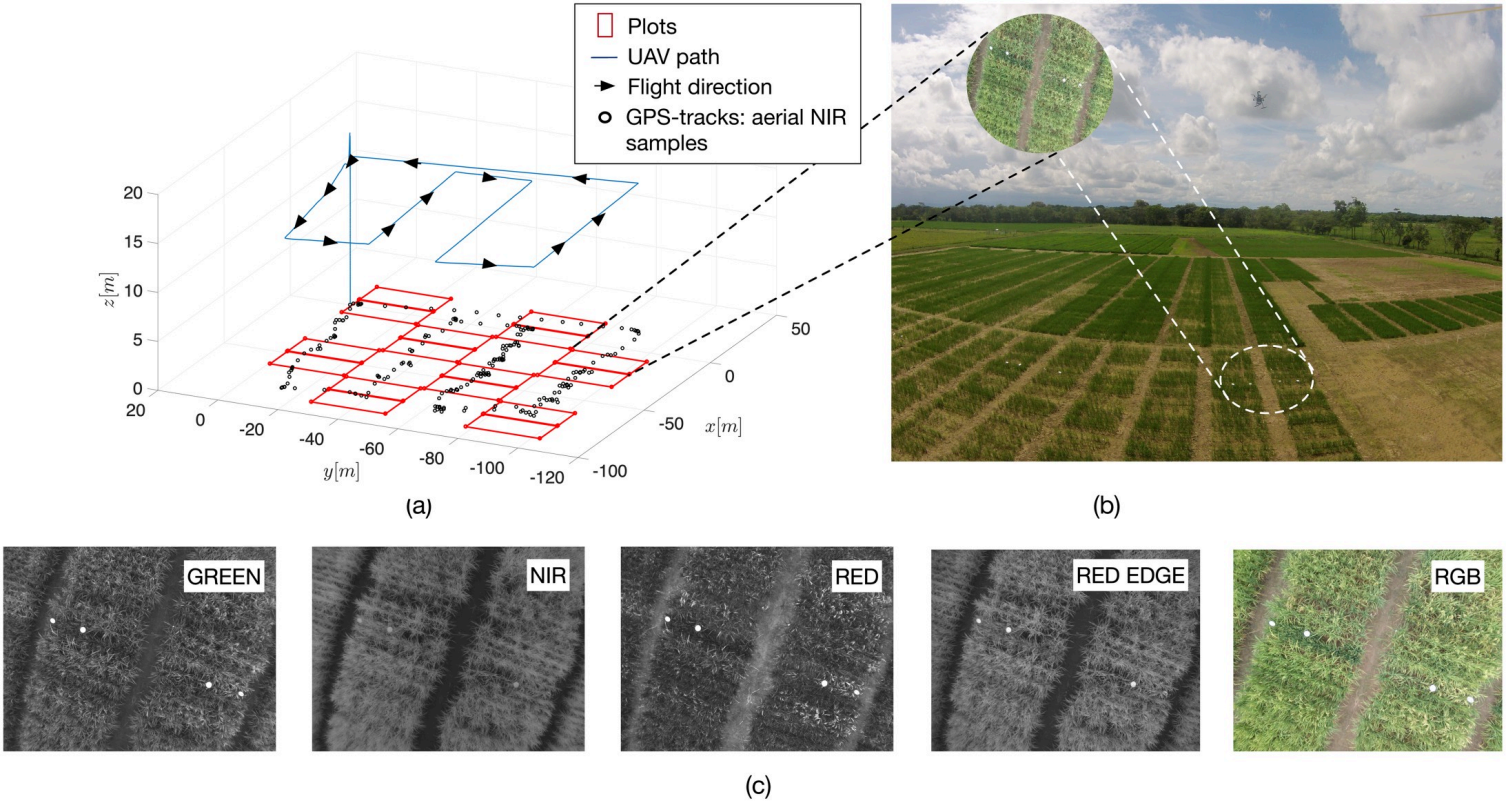
UAV crop coverage: (a) 3D flight trajectory. The UAV was set to fly at 20*m* over the crop at a maximum speed of 1.5*ms*^−1^. The black dots at ground-level correspond to the GPS-tracks of aerial imagery samples. (b) Crop fields—CIAT base station. (c) Parrot-Sequoia multispectral camera bands.

Fig 11(a) details how the crop data was captured. The UAV was programmed to cover the crop by following GPS-waypoints at a constant altitude of 20*m* above the crop, with a maximum linear velocity of 1.5*ms*^−1^. The parrot Sequoia multispectral camera offers a resolution of 1280 × 960 for each independent spectral sensor, yielding a crop-to-image resolution of 1.83cm/pixel according to the flying altitude. At each waypoint, the UAV hovers during 3*s* to capture geo-referenced NIR images of the plot of interest. As shown by Fig 11b and 11c, all the images were registered to match with the positions of the ground-level markers. The Parrot Sequoia camera was equipped with GPS, IMU+magnetometer and a solar radiation sensor enabling geo-tagging, image perspective correction and the regulation of the amount of absorbed light, respectively. As a result, all the images generated by each independent band (green, red, red-edge and near-infrared) were automatically compensated for changing weather conditions and canopy reflections.

**Fig 12:**
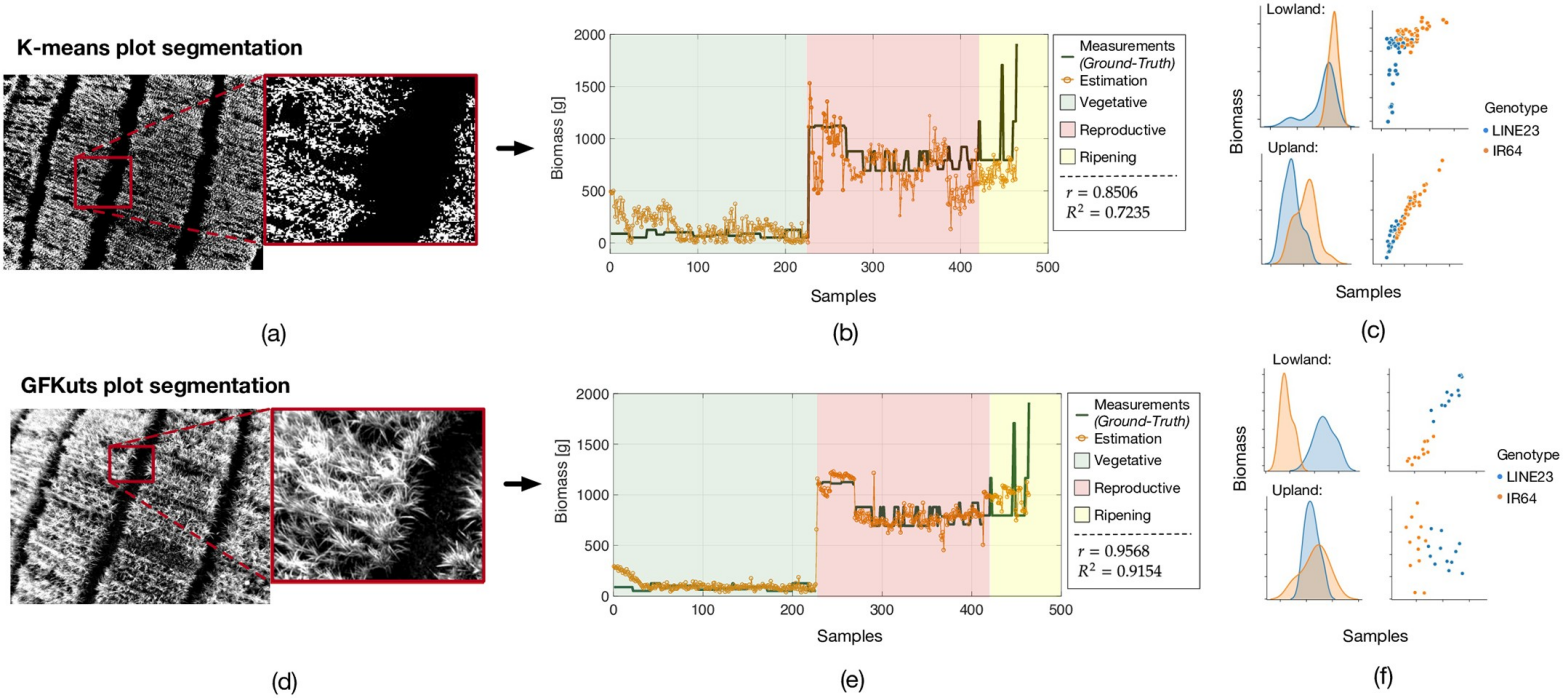
AGBD estimation results: (a,d) plot segmentation comparative results between the K-means and the proposed GFKuts approach. (b,e) ANN-driven estimations in biomass VS Ground-truth measurements. (c,f) ElasticNet-driven identification of the biomass readings according to the planted rice varieties under lowland and upland production systems.

Now, in Fig 12 we compare the impact that the applied segmentation method plays for an accurate biomass estimation. In turn, the upper plots (a-c) show the results of applying a standard K-means segmentation approach, whereas the lower plots (d-f) show the AGBD estimation results achieved by the proposed GFKuts method introduced in Section 2. As shown in plot (d), the GFKuts approach achieved smooth pixel information with richer detail of the canopy structure, enabling the accurate segmentation of the NIR imagery acquired. After the segmentation, the seven VI-features introduced in Table 2 are extracted from the images. Given that, plots (b) and (e) show the results of the Artificial Neural Networks (ANN) trained with the selected VIs to predict the biomass dynamics during the entire crop phenological stages. In both cases, we used ANNs with one hidden layer composed by 15 neurons and the Levenberg-Marquardt non-linear training function. This configuration was selected according to the findings previously reported in [29], where strong non-linear dependencies between the vegetation indices with the biomass variations were found through the phenological cycle, concretely, when the rice plants began to senesce, making the yellow color of the plants predominant. On the other hand, deep-learning methods such as Convolutional Neural Networks (CNN), tend to require more computational time during the pooling through lots of hidden layers in order to detect data features. For this application, we use well-known Vegetative Index features that have been widely used and validated in the specialized literature [3, 4, 7]. Other image-based features such as color, structure, and morphology do not work well with low-resolution multispectral imagery. In fact, the main advantage of using Vegetative Indices (as features for training), relies on having information from several light reflectances at different wavelengths, providing key information of the plant health status and variables.

As shown in Fig 12(e), a significant improvement was obtained for the AGBD estimation based on the GFKuts-driven input data. The performance was measured in terms of the linear correlation (*r*) and the coefficient of determination (*R*^2^). On average, we obtained an AGBD correlation of 0.9568 with *R*^2^ = 0.9154, increasing the estimation in about 13%. The samples-axis in both figs (b) and (e) correspond to the aerial imagery used for the estimation of biomass thought the phenological cycle. As previously mentioned in Section 2, biomass destructive measurements were conducted for selected crop plots, in order to assemble the Ground-Truth dataset. Given that, our system selects those aerial samples matching with the GPS coordinates of the ground measurements.

Lastly, Fig 12c–12f present the comparative results of both approaches for the estimation of the AGBD according to the physiological response of two rice genotypes under lowland and upland production systems. Elastic-Net regressions [43] were used to determine these relationships. This method overcomes several limitations of standard multi-variable regressions by combining the penalties of both lasso and ridge regression methods, with the aim of minimizing the following loss function:

$$\begin{array}{c} \begin{array}{c} L_{net}\left(\beta \right)=MSE+r\alpha \sum_{i=1}^{n}\left|\beta_{i}\right|+\frac{1-r}{2}\alpha \sum_{i=1}^{n}\beta_{i}^{2}, \end{array} \end{array}$$ (5)

where *r* is the mixing parameter between ridge *r* = 0 and lasso *r* = 1. The MSE term is the mean squared error, while *α* enables the regularized regression for the penalty function. Eq 5 was trained with two different Ground-truth data; one for lowland and the other for upland. As observed in plot (f), we obtained an accurate separability for lowland, but it still remains challenging to identify the biomass readings for each rice variety in upland systems. This could be happening due to both varieties has an inversely proportional relation between biomass production and plant stature, e.g. although Line23 is adapted to upland rice cultivation, IR64 tends to produce more biomass with a shorter stature, whereas Line23 is exactly the opposite. In upland (dry soils), concretely after the reproductive stage, it is more difficult to distinguish in between varieties since the differences among biomass accumulation, plant stature and soil noise are barely detected. In lowland (flooded paddies), the water layer facilitates the segmentation process, which in turn, reduces the background noise.

## 4 Conclusions

The proposed plot segmentation approach (GFKuts) enabled the precise characterization of Vegetative Index (VI) features, by associating different spectral reflectances with smooth pixel information and richer detail of the canopy structure. This segmentation process was fully automatic thanks to the Montecarlo-sampled K-means integration and it can run embedded on board the UAV’s computational kernel. Our segmentation method was applied by combining pixel information from four channels, in which the Red-Green-NIR (RGN) and the red-edge color space enabled the most accurate and fine identification of the canopy cluster. It is important to highlight that most of the existing body of work in image processing for crop phenotyping apply traditional thresholding, histograms or clustering methods for plot segmentation.

On average, we obtained an Above-Ground Biomass correlation of 0.9568 with *R*^2^ = 0.9154, increasing the estimation in about 13% compared to the standard K-means approach (cf. Fig 12e). Neural network models were trained with the extracted VIs by including both time-independent imagery samples and time-dependent VI dynamics i.e, the evolution of the features over time, as shown by Fig 10c, while imagery was captured with a sampled frequency of 2*Hz*.

In addition, as the biomass increased and the plants began to senesce, panicles also appeared during the reproductive and ripening stages (cf. Fig 3). We found these changes in the canopy make it difficult to associate the extracted VIs with the canopy’s biomass, therefore decreasing both correlation and coefficient of determination for the final crop stages. Although some VI features did not saturate for higher values of biomass (e.g. CTVI), they neither provided a precise estimation of the biomass during the ripening stage, since their correlation with D-BM and F-BM was lower, as seen on Fig 9. In fact, this is the reason why our methods rely on the combination of several VIs, allowing an accurate biomass estimation regardless of the crop production system (lowland or upland).

Future work will focus on the characterization of new VIs that do not saturate at higher values of biomass, and incorporate other morphological features at the plant-scale required to enhance the training dataset for the machine learning models.

## Supporting information

S1 Video The video is available as supporting material in the online version of this article. The video accompanying this paper illustrates the steps performed during the experiments.
(MP4)

S1 File RAW data supporting image segmentation metrics, NIR imagery used for Machine Learning testing, and biomass estimation results. Available at the Open Science Framework: https://osf.io/cde6h/?view_only=1c4e5e03b9a34d3b96736ad8ab1b2774.
(ZIP)

S2 File Experimental protocol for crop monitoring. Available at: https://www.protocols.io/view/protocol-bjxskpne.
(PDF)
