# Supplementary material for: A novel NIR-image segmentation method for the precise estimation of above-ground biomass in rice crops
Source: PLoS One. 2020 Oct 5;15(10):e0239591. doi: 10.1371/journal.pone.0239591 (PMC7535130; doi:10.1371/journal.pone.0239591)
Supplement: S2 File — Available at: https://www.protocols.io/view/protocol-bjxskpne. (PDF) [file pone.0239591.s003.pdf]

OMICAS P4: DISTRIBUTED PLATFORM FOR HIGH-RESOLUTION MULTISCALE PHENOTYPING OF CROPS

Project:	61187 - OMICAS: IN-SILICO MULTISCALE OPTIMIZATION OF SUSTAINABLE AGRICULTURAL CROPS (ÓMICAS)	
Doc-No.	P4.FR#1-12_2018	
Rev-No.	2.0	
Date:	05/01/2019	
Author(s):	Eliel Petro<br>Camila Rebolledo<br>Julián Colorado	
Institutions:	Centro Internacional de Agricultura Tropical -CIAT<br>Pontificia Universidad Javeriana Bogotá	
Prepared by:	Eliel Petro, Camila Rebolledo	Fdo. ____________________
Revised by:	Julián Colorado<br>Diego Méndez<br>Iván F. Mondragón	Fdo. ____________________
		Date.

TABLE OF CONTENTS

1) Field Experiments Description 2

2) Experiments for different densities, and nitrogen and water doses 2
Experiments 4, 5, 6: Palmira – Valle (July – November 2018) 2
3) Validation experiments and evaluation of 330 varieties 6
Experiment 7: Palmira – Valle (August – December 2018) 6

APPENDIX 4: EXPERIMENT 4: PALMIRA – VALLE WITH 200 KG OF NITROGEN AND NO IRRIGATION FROM VEGETATIVE TO RIPENING STAGES (JULY – NOVEMBER 2018) 7

APPENDIX 5: EXPERIMENT 5: PALMIRA – VALLE WITH 200 KG OF NITROGEN AND CONTINUOUS WATERING (JULY – NOVEMBER 2018) 10

APPENDIX 6: EXPERIMENT 6: PALMIRA – VALLE WITH 100 KG OF NITROGEN AND CONTINUOUS IRRIGATION (JULY – NOVEMBER 2018) 13

APPENDIX 7: EXPERIMENT 7: PALMIRA – VALLE (AUGUST – DECEMBER 2018) 16

REPORT

1) Field Experiments Description

- **Method Validation Experiment:** the images will be taken in 6 different phases of the crop: germination, maximum tillering, panicle start, flowering, 10 days after flowering and harvesting. Simultaneously, 1m linear samples will be taken per plot.
- **Different Densities Experiments:** these experiments will be applied in a 700 m^2 area, evaluating 25 lines in 3 m^2 plots with 3 repetitions. These 25 lines are CIAT elite lines which are part of the rice improvement program and from farms of the Tolima's Fedearroz.

2) Experiments for different densities, and nitrogen and water doses

Experiments 4, 5, 6: Palmira – Valle (July – November 2018)

The genetic material that was used consisted in 6 very different genotypes in terms of cumulative dry biomass and vegetative cycle (Table 5) (including Line 23 and IR64). The reference range for cumulative dry biomass (stems and leaves) and total dry biomass (stems, leaves and panicles) in previous experiments where Line 23 and IR64 were evaluated showed a variation of 15.56 to 3650 g m^2 of dry biomass. With this genotype biodiversity, a goal was to have a gradient in the cumulative dry mass and for the algorithm for dry biomass estimation to be able to detect the differences between genotypes and phenological stages.

Table 1: Relation between days to blooming and range of cumulative dry biomass for the selected genotypes.

Genotypes	Blooming 50% (Days)	Celsius degrees day (°C day)	Range of dry biomass (g m^{-2})		Range of total dry biomass (g m^{-2})	
			Minimum	Maximum	Minimum	Maximum
**FED50**	101	1380	539.22	1264.59	683.58	2632.40
**MG2**	97	1333	530.01	1309.16	625.36	3107.27
**ELWEE**	96	1305	539.51	2330.61	751.01	2579.76
**NORUNKAN**	97	1305	567.72	1469.95	647.65	2765.41
**IR64**	98	1243	71.69	2325.00	71.69	3650.00
**AZUCENA**	94	1215	625.84	959.22	791.00	1908.05
**UPLRI7**	87	1198	563.79	1790.96	1013.87	2594.98
**LÍNEA 23**	86	1100	15.56	850.00	15.56	1362.00

Dry biomass (stems and leaves), Total dry biomass (stems, leaves and panicles)

Experiments 4, 5 and 6 were established following a random complete block design with 3 repetitions and 8 genotypes for each repetition. Each experimental unit had an area of 5.77 m^2 (2.10 m x 2.75 m) and had 7 furrows (Figure 7). The sowing was performed using the transplanting method with a distance of 0.3m between furrows and 0.25m between plants, with 77 plants in each plot. Each sampling unit was composed by 4 plants by linear meter. The sowing and transplanting of the genotypes was interleaved (Table 6), starting with the sowing of the genotype with the highest cumulative degrees day and finalizing with the one with lowest cumulative value; all this was done looking to synchronize the reproductive and ripening stages and for the samples to be homogeneous in terms of the development of the genotype.

Figure 1: Plot design and sample areas in Palmira – Valle (July – November 2018).

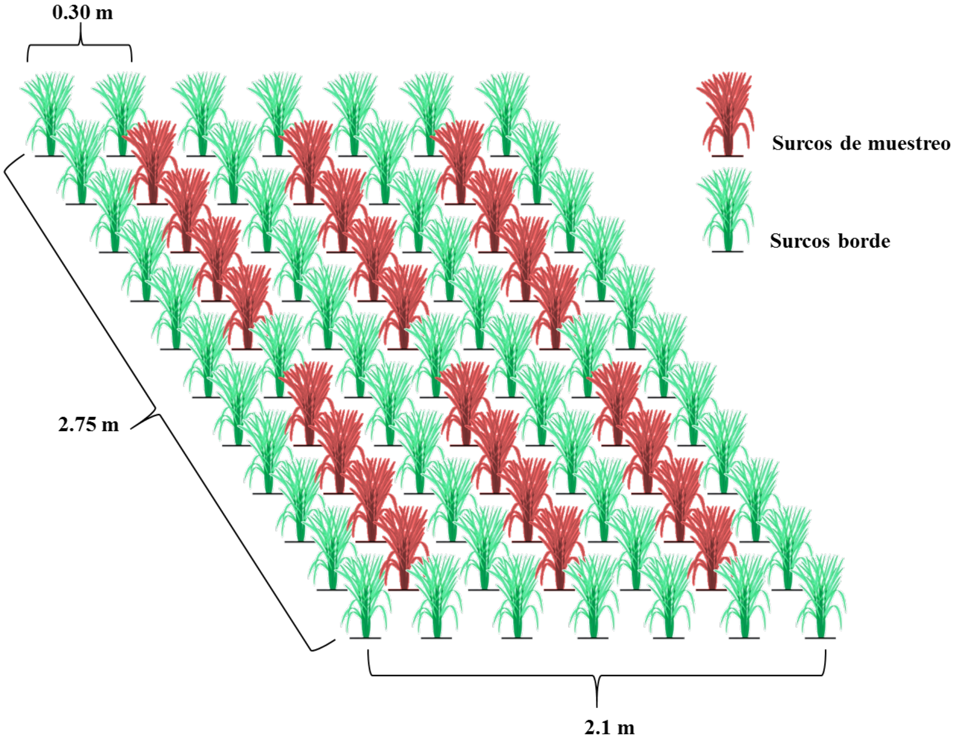

Table 2: Sowing sequence for the selected genotypes in experiments 4, 5 and 6 in Palmira – Valle (July – November 2018).

Sequence	Genotype	Sowing date	Emergency date	Transplanting date
**1**	**FED50**	07/05/2018	07/09/2018	08/03/2018
**2**	**MG2**	07/09/2018	07/13/2018	08/07/2018
**3**	**ELWEE**	07/10/2018	07/14/2018	08/08/2018
**4**	**NORUNKAN**	07/11/2018	07/16/2018	08/10/2018
**5**	**IR64**	07/14/2018	07/20/2018	08/14/2018
**6**	**AZUCENA**	07/16/2018	07/21/2018	08/15/2018
**7**	**UPLRI7**	07/18/2018	07/24/2018	08/18/2018
**8**	**LÍNEA 23**	07/23/2018	07/29/2018	08/23/2018

Experiments 4, 5 and 6 showed a variation in terms of fertilizer doses and the availability of irrigation:

- Experiment 4: 200kg of nitrogen and no irrigation from vegetative to ripening stages (Figure 8).
- Experiment 5: 200kg of nitrogen and continuous irrigation (Figure 9).
- Experiment 6: 100kg of nitrogen and continuous irrigation (Figure 10).

To evaluate the effect of irrigation limitation from vegetative to ripening stages for experiment 4, the leaf's temperature was evaluated utilizing a MultispeQ (also applicable for experiments 5 and 6).

Figure 2: Experiment 4: Palmira – Valle with 200 kg of nitrogen and no irrigation vegetative to ripening stages (July – November 2018).

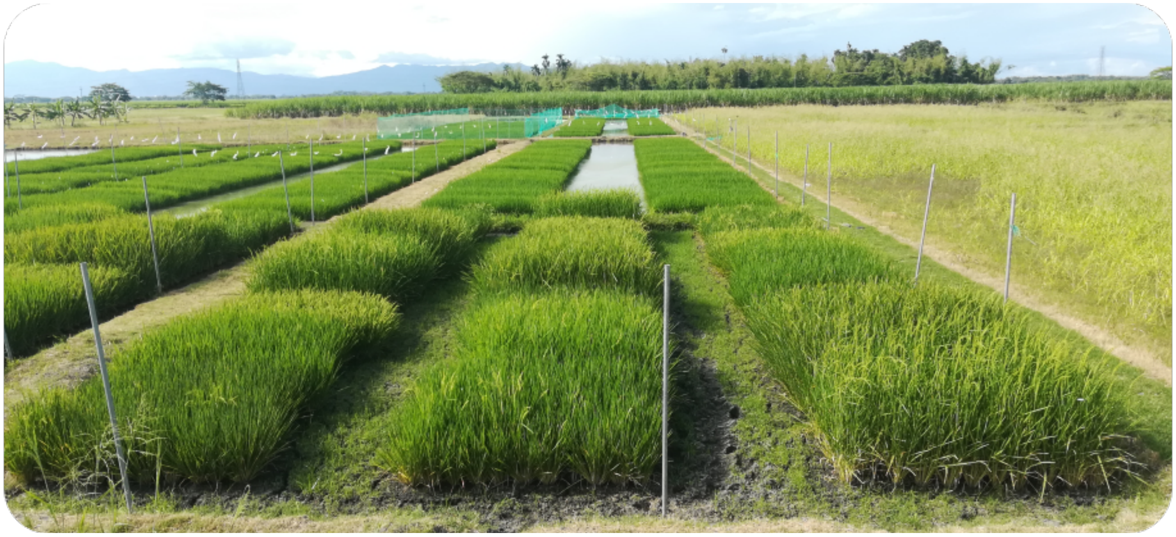

Figure 3: Experiment 5: Palmira – Valle with 200 kg of nitrogen and continuous irrigation (July – November 2018).

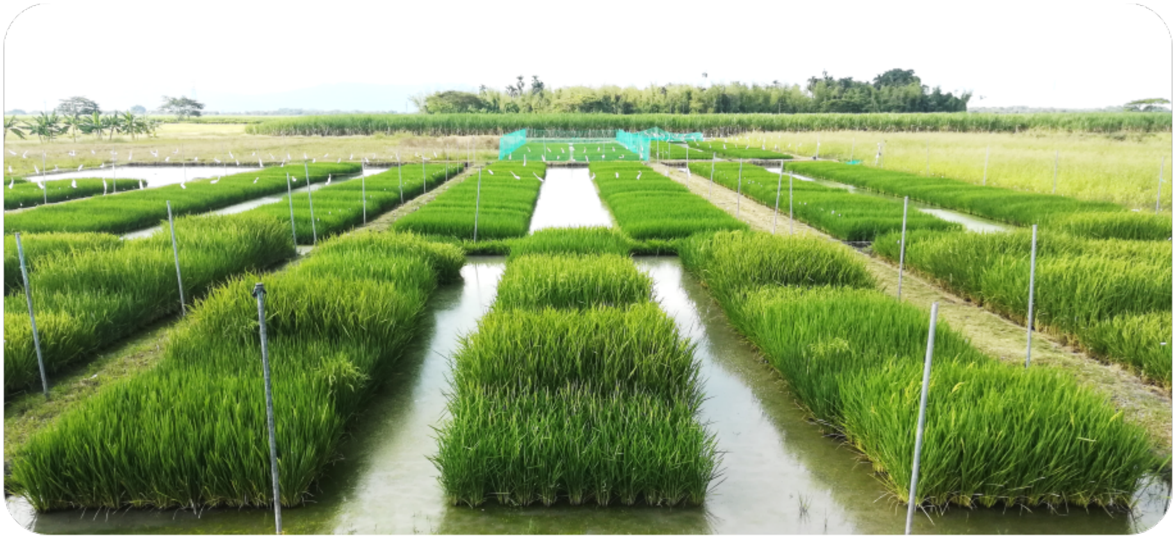

Figure 4: Experiment 6: Palmira – Valle with 100 kg of nitrogen and continuous irrigation (July – November 2018).

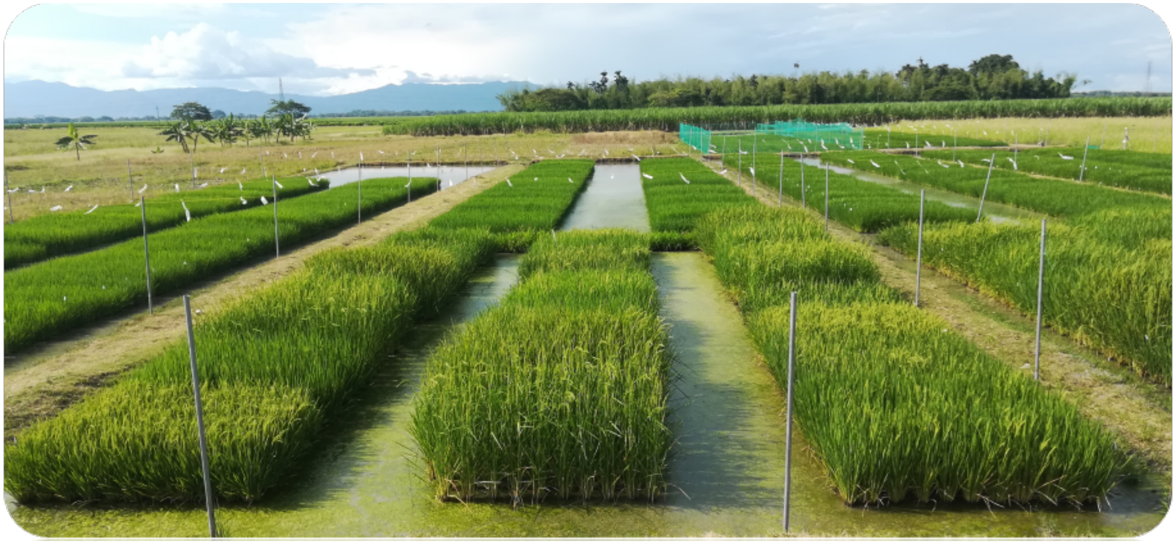

The fertilization plan for experiments 4, 5 and 6 are presented in Table 3, Table 4 and Table 5.

Table 3: Fertilization plan in Palmira – Valle with 200 kg of nitrogen and no irrigation from vegetative to ripening stages (August – December 2018).

**Element**	**Element dose (kg ha^{-1})**	**When transplanting**		**5 DAT**		**10 DAT**		**40 DAT**	
		**%**	**Dose**	**%**	**Dose**	**%**	**Dose**	**%**	**Dose**
**N**	**200.5**	0.0	0.0	20.0	44.5	50.0	111.2	30.0	66.7
**P_2O_5**	**59.8**	100.0	75.1	0.0	0.0	0.0	0.0	0.0	0.0
**K_2O**	**132.0**	50.0	63.5	0.0	0.0	50.0	63.5	0.0	0.0
**Zn**	**7.0**	100.0	16.2	0.0	0.0	0.0	0.0	0.0	0.0
**Fe**	**21.7**	100.0	65.8	0.0	0.0	0.0	0.0	0.0	0.0

Table 4: Fertilization plan in Palmira – Valle with 200 kg of nitrogen and continuous irrigation (August – December 2018).

**Element**	**Element dose (kg ha^{-1})**	**When transplanting**		**5 DAT**		**10 DAT**		**40 DAT**	
		**%**	**Dose**	**%**	**Dose**	**%**	**Dose**	**%**	**Dose**
**N**	**200.5**	0.0	0.0	20.0	44.5	50.0	111.2	30.0	66.7
**P_2O_5**	**59.8**	100.0	75.1	0.0	0.0	0.0	0.0	0.0	0.0
**K_2O**	**132.0**	50.0	63.5	0.0	0.0	50.0	63.5	0.0	0.0
**Zn**	**7.0**	100.0	16.2	0.0	0.0	0.0	0.0	0.0	0.0
**Fe**	**21.7**	100.0	65.8	0.0	0.0	0.0	0.0	0.0	0.0

Table 5: Fertilization plan in Palmira – Valle with 100 kg of nitrogen and continuous irrigation (August – December 2018).

Element	Element dose (kg ha^{-1})	When transplanting		5 DAT		10 DAT		40 DAT	
		%	Dose	%	Dose	%	Dose	%	Dose
**N**	**200.5**	0.0	0.0	20.0	19.3	50.0	48.2	30.0	28.9
**P_2O_5**	**59.8**	100.0	75.1	0.0	0.0	0.0	0.0	0.0	0.0
**K_2O**	**132.0**	50.0	63.5	0.0	0.0	50.0	63.5	0.0	0.0
**Zn**	**7.0**	100.0	16.2	0.0	0.0	0.0	0.0	0.0	0.0
**Fe**	**21.7**	100.0	65.8	0.0	0.0	0.0	0.0	0.0	0.0

The weed control consisted in the usage of the herbicides Butaclor (4 L ha^{-1}) and Basagran (3 L ha^{-1}) before transplanting, while for insect control Regent (0.3 L ha^{-1}) was used. No diseases were detected.

During the experiments, pictures were taken at the start of the vegetative stage and 3 samples: one sample during blooming and two during maturity stages, with their corresponding pictures. The results of the samples and measurements are included in **Appendix 4** (experiment 4), **Appendix 5** (experiment 5) and **Appendix 6** (experiment 6).

3) Validation experiments and evaluation of 330 varieties

Experiment 7: Palmira – Valle (August – December 2018)

The seventh experiment was carried out in order to validate the algorithm, in terms of dry matter content, water content and nitrogen content in a population of 330 contrasting genotypes. This experiment has focused on the flowering cycle, cumulative dry biomass, leaf coloration and modifying the sowing density. This experiment was established under a partially replicated (p-rep) design. Each experimental unit had an area of 4 m^2 (4.0 m x 1.0 m) and had 5 furrows (Figure 5). The sowing was performed using the transplanting method with a distance of 0.20 m between furrows and 0.20 m between plants, with 100 plants per plot (Figure 12)

For this experiment, images were taken with multispectral cameras 94 days after sowing, when the first genotypes began flowering. Afterwards, three samplings were performed including their respective images. 48 of the 330 genotypes were harvested. These will be used to train the algorithm developed by the Javeriana University. The remaining 282 genotypes will be used for validation. The results of the samples and measurements are included in **Appendix 7** (experiment 7).

Figure 5: Plot design in Palmira - Valle (August - December 2018)

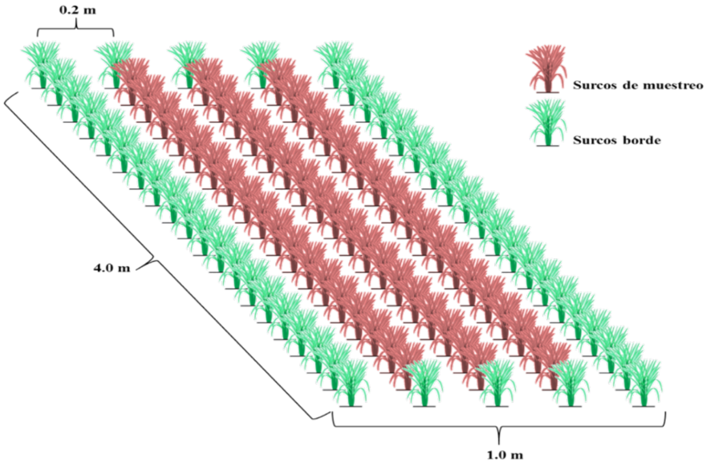

Figure 6: Experiment 7: Palmira – Valle (August - December 2018)

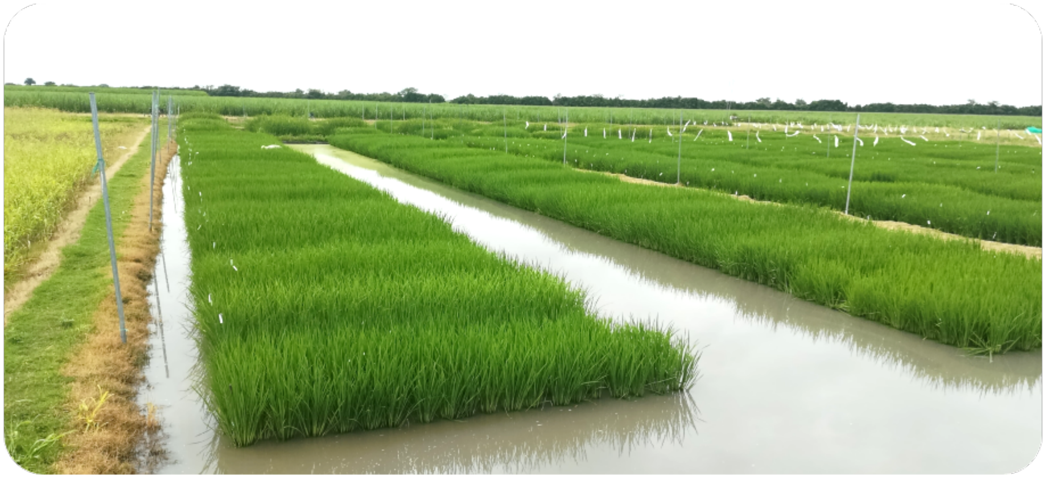

The fertilization plan for the genotypes in Palmira - Valle is presented in Table 10 in days after transplantation (DAT)

Table 6: Fertilization plant in Palmira – Valle (August – December 2018).

Element	Element dose (kg ha^{-1})	When transplanting		5 DAT		10 DAT		40 DAT	
		%	Dose	%	Dose	%	Dose	%	Dose
**N**	**200.5**	0.0	0.0	20.0	30.8	50.0	77.0	30.0	46.2
**P_2O_5**	**59.8**	100.0	52.0	0.0	0.0	0.0	0.0	0.0	0.0
**K_2O**	**132.0**	50.0	44.0	0.0	0.0	50.0	44.0	0.0	0.0
**Zn**	**7.0**	100.0	11.2	0.0	0.0	0.0	0.0	0.0	0.0
**Fe**	**21.7**	100.0	45.6	0.0	0.0	0.0	0.0	0.0	0.0

Values in grams applied correspond to the fertilizers: urea, diammonium phosphate, potassium chloride, zinc sulfate and iron sulfate.

The weed control consisted in the usage of the herbicides Butaclor (4 L ha^{-1}) and Basagran (3 L ha^{-1}) before transplanting, while for insect control Regent (0.3 L ha^{-1}) was used. No diseases were detected.

APPENDIXES

Appendix 4: Experiment 4: Palmira – Valle with 200 kg of nitrogen and no irrigation from vegetative to ripening stages (July – November 2018)

Table 7: Sampling 1 (10/01/2018) of dry biomass and SPAD reading in Palmira - Valle.

Plot	Genotype	Rep	Date	Plants fresh biomass (gr)	Plants Dry biomass (gr)	Hydric content (gr)	Panicle dry biomass (gr)	SPAD reading	Leaf temperature (ºC)
**1**	**AZUCENA**	1	10/01/2018	-	-	-	-	63.50	26.61
**2**	**ELWEE**	1	10/01/2018	-	-	-	-	48.20	27.72

**3**	**LÍNEA 23**	1	10/01/2018	-	-	-	-	57.08	28.01
**4**	**UPLRI7**	1	10/01/2018	-	-	-	-	47.89	29.94
**5**	**NORUNKAN**	1	10/01/2018	-	-	-	-	44.80	25.82
**6**	**IR64**	1	10/01/2018	-	-	-	-	52.32	29.31
**7**	**FED50**	1	10/01/2018	-	-	-	-	55.11	30.11
**8**	**MG2**	1	10/01/2018	-	-	-	-	52.14	29.83
**9**	**AZUCENA**	2	10/01/2018	-	-	-	-	62.79	31.62
**10**	**IR64**	2	10/01/2018	-	-	-	-	53.45	29.77
**11**	**LÍNEA 23**	2	10/01/2018	-	-	-	-	61.70	31.56
**12**	**UPLRI7**	2	10/01/2018	-	-	-	-	49.32	29.71
**13**	**ELWEE**	2	10/01/2018	-	-	-	-	55.01	30.09
**14**	**FED50**	2	10/01/2018	-	-	-	-	58.68	29.85
**15**	**NORUNKAN**	2	10/01/2018	-	-	-	-	49.86	29.50
**16**	**MG2**	2	10/01/2018	-	-	-	-	50.30	30.55
**17**	**FED50**	3	10/01/2018	-	-	-	-	57.67	29.74
**18**	**UPLRI7**	3	10/01/2018	-	-	-	-	49.99	30.31
**19**	**IR64**	3	10/01/2018	-	-	-	-	52.58	30.28
**20**	**AZUCENA**	3	10/01/2018	-	-	-	-	63.30	29.24
**21**	**NORUNKAN**	3	10/01/2018	-	-	-	-	52.53	29.06
**22**	**ELWEE**	3	10/01/2018	-	-	-	-	53.59	29.29
**23**	**LÍNEA 23**	3	10/01/2018	-	-	-	-	60.78	30.40
**24**	**MG2**	3	10/01/2018	-	-	-	-	53.96	31.00
**Mean**								**54.44**	**29.56**

Table 8: Sampling 2 (10/25/2018) of dry biomass and SPAD reading in Palmira - Valle.

Plot	Genotype	Rep	Date	Plants fresh biomass (gr)	Plants Dry biomass (gr)	Hydric content (gr)	Panicle dry biomass (gr)	SPAD reading	Leaf temperature (ºC)
**1**	**AZUCENA**	1	10/25/2018	1478.00	330.00	77.67	36.39	56.89	27.41
**2**	**ELWEE**	1	10/25/2018	1818.00	380.00	79.10	67.73	49.28	27.80
**3**	**LÍNEA 23**	1	10/25/2018	722.00	166.00	77.01	21.18	55.35	28.14
**4**	**UPLRI7**	1	10/25/2018	1528.00	348.00	77.23	57.94	44.63	27.89
**5**	**NORUNKAN**	1	10/25/2018	1194.00	272.00	77.22	44.68	40.71	28.50
**6**	**IR64**	1	10/25/2018	1334.00	304.00	77.21	86.17	47.84	27.97
**7**	**FED50**	1	10/25/2018	1454.00	347.94	76.07	54.34	47.32	29.07
**8**	**MG2**	1	10/25/2018	1552.00	359.30	76.85	46.24	44.66	26.77
**9**	**AZUCENA**	2	10/25/2018	1320.00	286.00	78.33	46.03	54.53	26.15
**10**	**IR64**	2	10/25/2018	1474.00	307.83	79.12	80.25	48.08	26.91
**11**	**LÍNEA 23**	2	10/25/2018	794.00	174.00	78.09	26.70	52.41	26.74
**12**	**UPLRI7**	2	10/25/2018	1632.00	342.00	79.04	73.53	47.31	28.82
**13**	**ELWEE**	2	10/25/2018	1634.00	346.00	78.82	60.14	48.82	27.42
**14**	**FED50**	2	10/25/2018	1248.00	304.00	75.64	60.99	51.99	26.75
**15**	**NORUNKAN**	2	10/25/2018	1304.00	304.00	76.69	48.38	43.04	25.08
**16**	**MG2**	2	10/25/2018	1556.00	346.87	77.71	47.63	50.42	27.22
**17**	**FED50**	3	10/25/2018	1318.00	303.48	76.97	34.12	45.99	29.54
**18**	**UPLRI7**	3	10/25/2018	1072.00	232.00	78.36	45.27	43.81	28.78
**19**	**IR64**	3	10/25/2018	1076.00	250.00	76.77	63.35	42.84	28.83
**20**	**AZUCENA**	3	10/25/2018	1116.00	226.00	79.75	39.56	56.73	27.55
**21**	**NORUNKAN**	3	10/25/2018	1402.00	296.00	78.89	53.63	45.98	27.30
**22**	**ELWEE**	3	10/25/2018	2054.00	409.35	80.07	86.01	45.51	29.78
**23**	**LÍNEA 23**	3	10/25/2018	824.00	170.47	79.31	25.78	50.93	28.72

**24**	**MG2**	3	10/25/2018	1516.00	334.28	77.95	48.72	44.36	26.23
**Mean**				**1350.83**	**297.48**	**77.91**	**52.28**	**48.31**	**27.72**

Table 9: Sampling 3 (11/08/2018) of dry biomass and SPAD reading in Palmira - Valle

Plot	Genotype	Rep	Date	Plants fresh biomass (gr)	Plants Dry biomass (gr)	Hydric content (gr)	Panicle dry biomass (gr)	SPAD reading	Leaf temperature (ºC)
**1**	**AZUCENA**	1	11/08/2018	1472.76	231.86	84.26	146.07	56.55	31.59
**2**	**ELWEE**	1	11/08/2018	1949.84	316.57	83.76	219.08	47.80	30.71
**3**	**LÍNEA 23**	1	11/08/2018	1032.36	178.53	82.71	100.77	54.55	30.81
**4**	**UPLRI7**	1	11/08/2018	1597.05	263.45	83.50	159.55	46.32	30.76
**5**	**NORUNKAN**	1	11/08/2018	1682.75	289.48	82.80	189.51	43.30	29.40
**6**	**IR64**	1	11/08/2018	1723.10	280.97	83.69	267.46	32.91	32.12
**7**	**FED50**	1	11/08/2018	1832.02	385.76	78.94	172.91	47.06	30.78
**8**	**MG2**	1	11/08/2018	1641.10	295.31	82.01	159.13	43.36	29.00
**9**	**AZUCENA**	2	11/08/2018	1281.67	220.11	82.83	173.98	49.26	28.70
**10**	**IR64**	2	11/08/2018	1577.04	273.77	82.64	205.70	42.59	32.62
**11**	**LÍNEA 23**	2	11/08/2018	875.57	159.50	81.78	85.28	49.82	32.67
**12**	**UPLRI7**	2	11/08/2018	1567.63	262.77	83.24	148.68	48.15	32.18
**13**	**ELWEE**	2	11/08/2018	1917.16	346.16	81.94	199.42	41.29	32.26
**14**	**FED50**	2	11/08/2018	1888.46	337.99	82.10	181.60	46.29	32.75
**15**	**NORUNKAN**	2	11/08/2018	1945.11	341.14	82.46	187.82	42.82	33.08
**16**	**MG2**	2	11/08/2018	2120.31	361.48	82.95	149.80	38.96	34.60
**17**	**FED50**	3	11/08/2018	1640.32	316.59	80.70	162.00	49.20	32.49
**18**	**UPLRI7**	3	11/08/2018	1590.09	282.80	82.21	187.69	40.88	30.47
**19**	**IR64**	3	11/08/2018	1760.36	286.99	83.70	253.02	40.23	30.18
**20**	**AZUCENA**	3	11/08/2018	1435.09	251.82	82.45	202.48	55.81	30.20
**21**	**NORUNKAN**	3	11/08/2018	1528.36	256.17	83.24	149.82	43.96	31.71
**22**	**ELWEE**	3	11/08/2018	2314.02	397.05	82.84	274.70	49.70	32.56
**23**	**LÍNEA 23**	3	11/08/2018	770.55	132.37	82.82	81.93	46.45	32.24
**24**	**MG2**	3	11/08/2018	2032.56	347.57	82.90	176.00	42.67	32.79
**Mean**				**1632.30**	**284.01**	**82.60**	**176.43**	**45.83**	**31.53**

Table 10: Sampling 4 (11/29/2018) of dry biomass and SPAD reading in Palmira - Valle

Plot	Genotype	Rep	Date	Plants fresh biomass (gr)	Plants D biomass (gr)	Hydric content (gr)	Panicle dry biomass (gr)	SPAD reading	Leaf temperature (ºC)
**1**	**AZUCENA**	1	11/29/2018	1075.70	266.49	75.23	238.59	33.06	28.45
**2**	**ELWEE**	1	11/29/2018	1191.85	306.07	74.32	229.16		
**3**	**LÍNEA 23**	1	11/29/2018	333.43	95.25	71.43	87.57	21.43	28.82
**4**	**UPLRI7**	1	11/29/2018	1327.01	297.40	77.59	301.52	19.77	28.59
**5**	**NORUNKAN**	1	11/29/2018	1250.93	260.74	79.16	253.50	24.11	28.66
**6**	**IR64**	1	11/29/2018	1019.18	224.35	77.99	249.84	15.86	30.10
**7**	**FED50**	1	11/29/2018	1446.36	350.82	75.74	205.72	31.13	29.80

**8**	**MG2**	1	11/29/2018	1331.24	290.93	78.15	248.53	23.03	30.92
**9**	**AZUCENA**	2	11/29/2018	1261.22	261.38	79.28	230.25	22.33	30.40
**10**	**IR64**	2	11/29/2018	1221.72	261.78	78.57	247.06	18.91	30.39
**11**	**LÍNEA 23**	2	11/29/2018	502.06	141.18	71.88	121.33		
**12**	**UPLRI7**	2	11/29/2018	1132.53	224.85	80.15	231.10	23.06	31.10
**13**	**ELWEE**	2	11/29/2018	1513.13	337.09	77.72	299.55		
**14**	**FED50**	2	11/29/2018	1528.80	359.77	76.47	223.42	32.86	30.69
**15**	**NORUNKAN**	2	11/29/2018	1276.65	269.55	78.89	263.03	24.24	30.28
**16**	**MG2**	2	11/29/2018	1063.26	239.03	77.52	247.49	26.50	31.08
**17**	**FED50**	3	11/29/2018	1510.45	334.26	77.87	242.42	31.19	29.72
**18**	**UPLRI7**	3	11/29/2018	1002.73	216.09	78.45	230.99	20.45	31.05
**19**	**IR64**	3	11/29/2018	1180.42	254.20	78.47	293.05	22.33	30.45
**20**	**AZUCENA**	3	11/29/2018	788.63	198.28	74.86	195.34		
**21**	**NORUNKAN**	3	11/29/2018	1380.22	298.32	78.39	270.80		
**22**	**ELWEE**	3	11/29/2018	1135.02	282.54	75.11	302.51		
**23**	**LÍNEA 23**	3	11/29/2018	702.98	187.11	73.38	166.44	22.75	31.32
**24**	**MG2**	3	11/29/2018	996.48	226.23	77.30	247.57	24.20	31.92
**Mean**				**1132.17**	**257.65**	**76.83**	**234.45**	**24.29**	**30.21**

Appendix 5: Experiment 5: Palmira – Valle with 200 kg of nitrogen and continuous watering (July – November 2018)

Table 11: Sampling 1 (10/01/2018) of dry biomass and SPAD reading in Palmira - Valle

Plot	Genotype	Rep	Date	Plants fresh biomass (gr)	Plants Dry biomass (gr)	Hydric content (gr)	Panicle dry biomass (gr)	SPAD reading	Leaf temperature (ºC)
**1**	**NORUNKAN**	1	10/01/2018	-	-	-	-	46.03	30.92
**2**	**FED50**	1	10/01/2018	-	-	-	-	56.49	30.15
**3**	**UPLRI7**	1	10/01/2018	-	-	-	-	45.40	30.87
**4**	**IR64**	1	10/01/2018	-	-	-	-	55.05	31.82
**5**	**ELWEE**	1	10/01/2018	-	-	-	-	55.16	31.92
**6**	**AZUCENA**	1	10/01/2018	-	-	-	-	64.30	31.81
**7**	**LÍNEA 23**	1	10/01/2018	-	-	-	-	60.05	32.21
**8**	**MG2**	1	10/01/2018	-	-	-	-	55.53	32.29
**9**	**NORUNKAN**	2	10/01/2018	-	-	-	-	50.94	33.08
**10**	**UPLRI7**	2	10/01/2018	-	-	-	-	43.11	32.63
**11**	**IR64**	2	10/01/2018	-	-	-	-	49.70	32.94
**12**	**MG2**	2	10/01/2018	-	-	-	-	55.32	33.14
**13**	**AZUCENA**	2	10/01/2018	-	-	-	-	63.51	32.95
**14**	**FED50**	2	10/01/2018	-	-	-	-	58.93	32.41
**15**	**ELWEE**	2	10/01/2018	-	-	-	-	47.65	32.69
**16**	**LÍNEA 23**	2	10/01/2018	-	-	-	-	58.22	33.15
**17**	**MG2**	3	10/01/2018	-	-	-	-	52.77	31.44
**18**	**FED50**	3	10/01/2018	-	-	-	-	58.53	31.34
**19**	**ELWEE**	3	10/01/2018	-	-	-	-	53.83	31.43
**20**	**UPLRI7**	3	10/01/2018	-	-	-	-	43.03	31.55
**21**	**NORUNKAN**	3	10/01/2018	-	-	-	-	48.87	31.45
**22**	**AZUCENA**	3	10/01/2018	-	-	-	-	59.82	30.85
**23**	**IR64**	3	10/01/2018	-	-	-	-	54.83	31.83

**24**	**LÍNEA 23**	3	10/01/2018	-	-	-	-	55.29	31.88
**Media**								**53.85**	**31.95**

Table 12: Sampling 2 (10/25/2018) of dry biomass and SPAD reading in Palmira - Valle

Plot	Genotype	Rep	Date	Plants fresh biomass (gr)	Plants Dry biomass (gr)	Hydric content (gr)	Panicle dry biomass (gr)	SPAD reading	Leaf temperature (ºC)
**1**	**NORUNKAN**	1	10/25/2018	1318.00	79.82	79.82	45.17	40.28	27.23
**2**	**FED50**	1	10/25/2018	1662.00	80.39	80.39	64.16	49.08	27.20
**3**	**UPLRI7**	1	10/25/2018	1480.00	81.22	81.22	49.19	43.24	28.24
**4**	**IR64**	1	10/25/2018	1706.00	82.88	82.88	74.50	46.08	28.78
**5**	**ELWEE**	1	10/25/2018	1616.00	78.09	78.09	59.01	47.81	27.93
**6**	**AZUCENA**	1	10/25/2018	1336.00	81.59	81.59	52.25	52.84	28.02
**7**	**LÍNEA 23**	1	10/25/2018	882.00	80.73	80.73	26.60	59.14	27.24
**8**	**MG2**	1	10/25/2018	1554.00	79.79	79.79	50.88	47.10	26.01
**9**	**NORUNKAN**	2	10/25/2018	1516.00	78.89	78.89	62.83	46.19	28.34
**10**	**UPLRI7**	2	10/25/2018	1904.00	83.18	83.18	52.30	48.76	27.10
**11**	**IR64**	2	10/25/2018	1622.00	81.87	81.87	79.68	50.70	28.50
**12**	**MG2**	2	10/25/2018	1930.00	81.66	81.66	48.04	49.49	30.25
**13**	**AZUCENA**	2	10/25/2018	1588.00	81.66	81.66	49.64	58.98	28.36
**14**	**FED50**	2	10/25/2018	1804.00	80.60	80.60	64.06	50.15	27.34
**15**	**ELWEE**	2	10/25/2018	2280.00	81.49	81.49	68.08	46.27	27.97
**16**	**LÍNEA 23**	2	10/25/2018	830.00	79.76	79.76	26.87	61.10	27.06
**17**	**MG2**	3	10/25/2018				59.67	46.80	28.02
**18**	**FED50**	3	10/25/2018	2602.00	83.66	83.66	59.97	49.97	29.36
**19**	**ELWEE**	3	10/25/2018	2116.00	82.61	82.61	73.85	44.57	27.80
**20**	**UPLRI7**	3	10/25/2018	1376.00	82.41	82.41	39.37	45.84	29.70
**21**	**NORUNKAN**	3	10/25/2018	1776.00	81.59	81.59	67.44	42.31	31.02
**22**	**AZUCENA**	3	10/25/2018	1744.00	82.11	82.11	54.90	56.43	29.92
**23**	**IR64**	3	10/25/2018	2204.00	84.15	84.15	95.31	46.50	28.96
**24**	**LÍNEA 23**	3	10/25/2018	996.00	79.72	79.72	29.98	61.66	29.45
**Mean**				**1645.30**	**307.97**	**81.30**	**56.41**	**49.64**	**28.33**

Table 13: Sampling 3 (11/10/2018) of dry biomass and SPAD reading in Palmira - Valle

Plot	Genotype	Rep	Date	Plants fresh biomass (gr)	Plants Dry biomass (gr)	Hydric content (gr)	Panicle dry biomass (gr)	SPAD reading	Leaf temperature (ºC)
**1**	**NORUNKAN**	1	11/10/2018	1921.36	327.20	82.97	189.32	40.11	29.73
**2**	**FED50**	1	11/10/2018	2051.21	332.48	83.79	176.49	47.01	29.83
**3**	**UPLRI7**	1	11/10/2018	1604.32	262.52	83.64	164.24	43.92	29.84
**4**	**IR64**	1	11/10/2018	1911.04	284.10	85.13	221.99	43.61	29.59
**5**	**ELWEE**	1	11/10/2018	1995.61	318.99	84.02	204.26	47.39	29.96
**6**	**AZUCENA**	1	11/10/2018	1544.02	264.60	82.86	146.70	50.65	28.95
**7**	**LÍNEA 23**	1	11/10/2018	624.81	169.99	72.79	102.12	54.03	29.72
**8**	**MG2**	1	11/10/2018	2078.45	329.30	84.16	208.44	43.85	29.20
**9**	**NORUNKAN**	2	11/10/2018	1947.49	310.10	84.08	199.40	39.76	29.12
**10**	**UPLRI7**	2	11/10/2018	1721.14	264.97	84.60	156.48	41.70	30.01
**11**	**IR64**	2	11/10/2018	1898.65	265.26	86.03	203.03	42.53	30.87

**12**	**MG2**	2	11/10/2018	1856.92	273.28	85.28	167.70	42.74	30.39
**13**	**AZUCENA**	2	11/10/2018	1632.08	250.78	84.63	158.93	51.39	31.50
**14**	**FED50**	2	11/10/2018	2402.90	349.50	85.46	192.73	46.14	30.74
**15**	**ELWEE**	2	11/10/2018	2304.73	355.29	84.58	215.15	43.67	32.30
**16**	**LÍNEA 23**	2	11/10/2018	928.45	155.85	83.21	85.82	56.38	31.46
**17**	**MG2**	3	11/10/2018	2021.76	329.19	83.72	171.18	41.01	31.63
**18**	**FED50**	3	11/10/2018	2338.70	386.06	83.49	196.99	52.87	31.69
**19**	**ELWEE**	3	11/10/2018	1838.98	293.90	84.02	200.47	45.70	31.91
**20**	**UPLRI7**	3	11/10/2018	1813.56	290.89	83.96	158.04	43.66	31.46
**21**	**NORUNKAN**	3	11/10/2018	1503.02	244.72	83.72	151.32	38.88	31.88
**22**	**AZUCENA**	3	11/10/2018	1597.04	278.08	82.59	181.69	46.63	30.38
**23**	**IR64**	3	11/10/2018	1653.26	248.16	84.99	193.93	40.30	30.27
**24**	**LÍNEA 23**	3	11/10/2018	1042.90	201.78	80.65	109.08	53.68	31.29
**Mean**				**1759.68**	**282.79**	**83.52**	**173.15**	**45.73**	**30.57**

Table 14: Sampling 4 (11/29/2018) of dry biomass and SPAD reading in Palmira - Valle

**Plot**	**Genotype**	**Rep**	**Date**	**Plants fresh biomass (gr)**	**Plants Dry biomass (gr)**	**Hydric content (gr)**	**Panicle dry biomass (gr)**	**SPAD reading**	**Leaf temperature (ºC)**
**1**	**NORUNKAN**	1	11/29/2018	1251.35	255.53	79.58	257.00	23.19	31.94
**2**	**FED50**	1	11/29/2018	1683.75	293.85	82.55	220.04	35.95	31.59
**3**	**UPLRI7**	1	11/29/2018	1457.99	247.39	83.03	269.33	23.54	31.79
**4**	**IR64**	1	11/29/2018	1597.78	262.09	83.60	280.36	26.70	32.28
**5**	**ELWEE**	1	11/29/2018	1615.25	293.57	81.83	284.06		
**6**	**AZUCENA**	1	11/29/2018	1242.96	248.28	80.03	221.32	43.72	32.50
**7**	**LÍNEA 23**	1	11/29/2018	531.84	138.60	73.94	127.88		
**8**	**MG2**	1	11/29/2018	1478.58	258.66	82.51	285.10	29.92	31.61
**9**	**NORUNKAN**	2	11/29/2018	1045.39	217.85	79.16	221.23	25.02	32.06
**10**	**UPLRI7**	2	11/29/2018	1430.02	246.38	82.77	253.89	25.07	32.64
**11**	**IR64**	2	11/29/2018	1693.13	267.67	84.19	298.48	27.04	32.64
**12**	**MG2**	2	11/29/2018	1689.41	280.55	83.39	269.20	31.43	32.94
**13**	**AZUCENA**	2	11/29/2018	1329.52	249.06	81.27	233.50	36.53	33.16
**14**	**FED50**	2	11/29/2018	1561.05	298.34	80.89	175.72	38.46	34.57
**15**	**ELWEE**	2	11/29/2018	1609.72	307.89	80.87	309.10		
**16**	**LÍNEA 23**	2	11/29/2018	525.30	155.87	70.33	127.25		
**17**	**MG2**	3	11/29/2018	1185.02	259.53	78.10	253.72	28.33	34.42
**18**	**FED50**	3	11/29/2018	1633.20	293.50	82.03	214.50	39.91	33.66
**19**	**ELWEE**	3	11/29/2018	1631.70	279.72	82.86	274.09		
**20**	**UPLRI7**	3	11/29/2018	1139.27	187.93	83.50	209.36	29.13	33.05
**21**	**NORUNKAN**	3	11/29/2018	1657.32	283.07	82.92	289.75	28.19	31.96
**22**	**AZUCENA**	3	11/29/2018	1134.22	236.12	79.18	192.40	37.02	33.19
**23**	**IR64**	3	11/29/2018	1628.12	240.18	85.25	257.30	30.41	31.80
**24**	**LÍNEA 23**	3	11/29/2018	753.64	145.63	80.68	131.88		
**Mean**				**1354.40**	**247.80**	**81.02**	**235.69**	**31.09**	**32.65**

Appendix 6: Experiment 6: Palmira – Valle with 100 kg of nitrogen and continuous irrigation (July – November 2018)

Table 15: Sampling 1 (10/01/2018) of dry biomass and SPAD reading in Palmira - Valle

Plot	Genotype	Rep	Date	Plants fresh biomass (gr)	Plants Dry biomass (gr)	Hydric content (gr)	Panicle dry biomass (gr)	SPAD reading	Leaf temperature (ºC)
**1**	**MG2**	1	10/01/2018	-	-	-	-	49.16	32.78
**2**	**FED50**	1	10/01/2018	-	-	-	-	53.77	31.98
**3**	**IR64**	1	10/01/2018	-	-	-	-	47.31	31.63
**4**	**LÍNEA 23**	1	10/01/2018	-	-	-	-	56.57	33.81
**5**	**AZUCENA**	1	10/01/2018	-	-	-	-	59.48	33.71
**6**	**UPLRI7**	1	10/01/2018	-	-	-	-	43.32	33.74
**7**	**NORUNKAN**	1	10/01/2018	-	-	-	-	47.97	33.38
**8**	**ELWEE**	1	10/01/2018	-	-	-	-	47.59	35.06
**9**	**IR64**	2	10/01/2018	-	-	-	-	50.88	33.18
**10**	**MG2**	2	10/01/2018	-	-	-	-	50.47	33.99
**11**	**FED50**	2	10/01/2018	-	-	-	-	56.28	34.05
**12**	**LÍNEA 23**	2	10/01/2018	-	-	-	-	61.04	34.32
**13**	**NORUNKAN**	2	10/01/2018	-	-	-	-	46.39	32.52
**14**	**UPLRI7**	2	10/01/2018	-	-	-	-	41.61	32.97
**15**	**ELWEE**	2	10/01/2018	-	-	-	-	50.48	32.43
**16**	**AZUCENA**	2	10/01/2018	-	-	-	-	56.41	32.72
**17**	**IR64**	3	10/01/2018	-	-	-	-	47.51	31.93
**18**	**UPLRI7**	3	10/01/2018	-	-	-	-	43.41	31.71
**19**	**AZUCENA**	3	10/01/2018	-	-	-	-	58.18	30.63
**20**	**NORUNKAN**	3	10/01/2018	-	-	-	-	44.64	31.97
**21**	**ELWEE**	3	10/01/2018	-	-	-	-	46.05	31.51
**22**	**FED50**	3	10/01/2018	-	-	-	-	54.76	31.34
**23**	**LÍNEA 23**	3	10/01/2018	-	-	-	-	58.45	31.54
**24**	**MG2**	3	10/01/2018	-	-	-	-	49.57	31.35
**Mean**								**50.89**	**32.68**

Table 16: Sampling 2 (10/25/2018) of dry biomass and SPAD reading in Palmira - Valle

Plot	Genotype	Rep	Date	Plants fresh biomass (gr)	Plants Dry biomass (gr)	Hydric content (gr)	Panicle dry biomass (gr)	SPAD reading	Leaf temperature (ºC)
**1**	**MG2**	1	10/25/2018	1526.00	310.06	79.68	41.51	44.13	29.42
**2**	**FED50**	1	10/25/2018	1340.00	284.00	78.81	56.95	46.08	31.76
**3**	**IR64**	1	10/25/2018	1606.00	296.00	81.57	101.30	43.84	32.07
**4**	**LÍNEA 23**	1	10/25/2018	894.00	172.00	80.76	18.80	58.99	31.10
**5**	**AZUCENA**	1	10/25/2018	1484.00	274.00	81.54	55.83	59.58	29.73
**6**	**UPLRI7**	1	10/25/2018	1504.00	298.00	80.19	53.54	44.30	30.08
**7**	**NORUNKAN**	1	10/25/2018	1488.00	290.00	80.51	62.27	45.07	30.71
**8**	**ELWEE**	1	10/25/2018	1828.00	329.50	81.97	78.55	48.84	31.29
**9**	**IR64**	2	10/25/2018	1376.00	288.00	79.07	62.74	48.55	27.12

**10**	**MG2**	2	10/25/2018	1466.00	302.00	79.40	46.93	47.22	25.56
**11**	**FED50**	2	10/25/2018	1256.00	270.00	78.50	49.13	47.62	29.56
**12**	**LÍNEA 23**	2	10/25/2018	976.00	224.00	77.05	20.90	58.97	25.84
**13**	**NORUNKAN**	2	10/25/2018	1222.00	240.16	80.35	40.12	44.29	26.89
**14**	**UPLRI7**	2	10/25/2018	1476.00	286.00	80.62	53.06	43.82	29.14
**15**	**ELWEE**	2	10/25/2018	1818.00	338.00	81.41	67.59	47.84	27.02
**16**	**AZUCENA**	2	10/25/2018	1290.00	240.00	81.40	54.79	53.75	27.54
**17**	**IR64**	3	10/25/2018	1560.00	262.00	83.21	79.18	44.27	31.28
**18**	**UPLRI7**	3	10/25/2018	1656.00	280.00	83.09	46.63	45.14	30.90
**19**	**AZUCENA**	3	10/25/2018	1282.00	238.32	81.41	54.34	56.23	30.07
**20**	**NORUNKAN**	3	10/25/2018	1476.00	272.00	81.57	65.92	45.34	28.98
**21**	**ELWEE**	3	10/25/2018	1394.00	248.00	82.21	60.99	38.53	30.82
**22**	**FED50**	3	10/25/2018	1280.00	238.08	81.40	31.02	47.06	29.46
**23**	**LÍNEA 23**	3	10/25/2018	972.00	202.00	79.22	29.01	57.98	27.81
**24**	**MG2**	3	10/25/2018	1448.00	298.00	79.42	42.70	43.45	27.92
**Mean**				**1400.75**	**270.01**	**80.60**	**53.08**	**48.37**	**29.25**

Table 17: Sampling 3 (11/10/2018) of dry biomass and SPAD reading in Palmira - Valle

Plot	Genotype	Rep	Date	Plants f biomass (gr)	Plants biomass	Hydric content (	Panicle biomass (	SPAD reading	Leaf temperature
**1**	**MG2**	1	11/10/2018	1663.32	264.12	84.12	195.37	38.09	30.97
**2**	**FED50**	1	11/10/2018	1600.45	259.40	83.79	189.82	43.36	30.01
**3**	**IR64**	1	11/10/2018	1299.04	192.19	85.21	187.96	37.65	30.52
**4**	**LÍNEA 23**	1	11/10/2018	957.09	167.90	82.46	72.27	54.65	31.63
**5**	**AZUCENA**	1	11/10/2018	1270.35	223.50	82.41	121.24	45.12	31.55
**6**	**UPLRI7**	1	11/10/2018	1610.23	255.09	84.16	147.70	43.39	31.89
**7**	**NORUNKAN**	1	11/10/2018	1553.78	248.81	83.99	168.50	39.05	31.32
**8**	**ELWEE**	1	11/10/2018	1671.68	253.19	84.85	223.29	42.84	31.38
**9**	**IR64**	2	11/10/2018	1542.86	247.43	83.96	210.99	36.93	31.06
**10**	**MG2**	2	11/10/2018	1700.60	288.50	83.04	143.71	36.12	31.62
**11**	**FED50**	2	11/10/2018	1554.86	247.93	84.05	162.22	43.80	32.24
**12**	**LÍNEA 23**	2	11/10/2018	740.12	136.03	81.62	76.15	53.93	33.41
**13**	**NORUNKAN**	2	11/10/2018	1617.18	265.60	83.58	194.67	40.14	32.85
**14**	**UPLRI7**	2	11/10/2018	1566.43	265.81	83.03	156.97	37.03	33.70
**15**	**ELWEE**	2	11/10/2018	2115.52	335.41	84.15	229.28	40.95	33.96

**16**	**AZUCENA**	2	11/10/2018	1304.15	218.97	83.21	152.40	46.38	33.37
**17**	**IR64**	3	11/10/2018	1627.92	234.73	85.58	180.17	36.12	33.24
**18**	**UPLRI7**	3	11/10/2018	1881.82	280.77	85.08	167.47	43.37	33.57
**19**	**AZUCENA**	3	11/10/2018	1361.43	212.93	84.36	145.73	44.82	33.92
**20**	**NORUNKAN**	3	11/10/2018	1472.52	231.96	84.25	148.97	38.35	33.82
**21**	**ELWEE**	3	11/10/2018	1847.48	266.83	85.56	208.86	44.99	34.10
**22**	**FED50**	3	11/10/2018	1664.42	262.04	84.26	155.98	46.86	32.96
**23**	**LÍNEA 23**	3	11/10/2018	665.88	131.68	80.22	70.43	51.44	32.64
**24**	**MG2**	3	11/10/2018	1537.40	250.18	83.73	170.21	37.52	32.26
**Mean**				**1492.77**	**239.21**	**83.78**	**161.68**	**42.62**	**32.42**

Table 18: Sampling 4 (11/29/2018) of dry biomass and SPAD reading in Palmira - Valle

**Plot**	**Genotype**	**Rep**	**Date**	**Plants fresh biomass (gr)**	**Plants Dry biomass (gr)**	**Hydric content (gr)**	**Panicle dry biomass (gr)**	**SPAD reading**	**Leaf temperature (ºC)**
**1**	**MG2**	1	11/29/2018	1115.51	208.14	81.34	203.70	18.57	31.93
**2**	**FED50**	1	11/29/2018	1495.60	243.86	83.69	232.09	35.93	32.41
**3**	**IR64**	1	11/29/2018	1114.35	173.19	84.46	206.91	20.45	32.82
**4**	**LÍNEA 23**	1	11/29/2018	613.32	131.47	78.56	103.77		
**5**	**AZUCENA**	1	11/29/2018	1141.32	194.83	82.93	175.83		
**6**	**UPLRI7**	1	11/29/2018	1532.72	244.15	84.07	235.06	22.58	35.68
**7**	**NORUNKAN**	1	11/29/2018	1345.03	201.80	85.00	224.22	34.58	35.47
**8**	**ELWEE**	1	11/29/2018	1168.52	188.97	83.83	219.73		
**9**	**IR64**	2	11/29/2018	1241.82	180.20	85.49	240.37	19.44	34.49
**10**	**MG2**	2	11/29/2018	1277.77	215.42	83.14	221.82	19.05	33.69
**11**	**FED50**	2	11/29/2018	1279.20	218.17	82.94	160.94	35.14	34.06
**12**	**LÍNEA 23**	2	11/29/2018	540.57	123.69	77.12	95.55		
**13**	**NORUNKAN**	2	11/29/2018	1340.38	241.95	81.95	241.62	19.96	33.04
**14**	**UPLRI7**	2	11/29/2018	1539.20	269.06	82.52	233.89	18.47	32.74
**15**	**ELWEE**	2	11/29/2018	1119.63	211.46	81.11	209.32		
**16**	**AZUCENA**	2	11/29/2018	1097.03	204.30	81.38	172.92	32.67	32.42
**17**	**IR64**	3	11/29/2018	1286.85	168.85	86.88	247.60	20.74	31.50
**18**	**UPLRI7**	3	11/29/2018	1159.52	182.56	84.26	206.92	16.84	31.24
**19**	**AZUCENA**	3	11/29/2018	1003.87	171.22	82.94	156.90	35.76	30.95
**20**	**NORUNKAN**	3	11/29/2018	1303.00	200.27	84.63	259.52	30.45	30.87
**21**	**ELWEE**	3	11/29/2018	938.01	166.92	82.20	199.75		
**22**	**FED50**	3	11/29/2018	1595.83	256.91	83.90	243.68	34.54	30.45
**23**	**LÍNEA 23**	3	11/29/2018	465.47	113.89	75.53	95.75		
**24**	**MG2**	3	11/29/2018	1033.72	175.96	82.98	189.94	21.94	29.94

**Mean**				**1156.18**	**195.30**	**82.62**	**199.08**	**25.71**	**32.57**

Appendix 7: Experiment 7: Palmira – Valle (August – December 2018)

Table 19: Sampling 1 (12/13/2018) of dry biomass and SPAD reading in Palmira - Valle

Plot	Genotype	Rep	Date	Plants fresh biomass (gr)	Plants Dry biomass (gr)	Hydric content (gr)	Panicle dry biomass (gr)	SPAD reading	Leaf temperature (ºC)
**2**	**G15**	1	12/13/2018	1201.81	173.62	85.55	193.37	38.21	26.85
**24**	**G86**	1	12/13/2018	1007.06	143.67	85.73	158.81	27.95	29.69
**34**	**G83**	1	12/13/2018	1155.02	160.49	86.11	204.28	36.87	28.26
**39**	**G175**	1	12/13/2018	889.45	115.45	87.02	132.42	30.70	31.44
**45**	**G184**	1	12/13/2018	1041.02	146.33	85.94	187.27	27.81	32.92
**57**	**G186**	1	12/13/2018	1108.68	158.69	85.69	155.69	37.39	29.92
**78**	**G247**	1	12/13/2018	1355.22	196.87	85.47	210.84	37.12	28.84
**128**	**G329**	1	12/13/2018	1101.04	139.19	87.36	205.20	25.99	30.52
**130**	**G264**	1	12/13/2018	1198.02	158.08	86.80	198.84	28.56	31.49
**143**	**G141**	1	12/13/2018	864.70	118.46	86.30	176.26	30.43	29.66
**165**	**G182**	1	12/13/2018	1079.92	155.16	85.63	175.60	35.27	28.26
**174**	**G12**	1	12/13/2018	1103.50	176.33	84.02	192.80	34.20	27.94
**210**	**G179**	1	12/13/2018	1167.20	160.79	86.22	173.70	39.46	27.44
**213**	**G1**	1	12/13/2018	844.10	126.27	85.04	128.46	24.73	29.22
**248**	**G35**	1	12/13/2018	1027.23	151.91	85.21	182.45		
**267**	**G315**	1	12/13/2018	1103.43	148.13	86.58	199.96	28.03	29.35
**Mean**				**1077.96**	**151.84**	**85.92**	**179.75**	**32.18**	**29.45**

Table 20: Sampling 2 (12/17/2018) of dry biomass and SPAD reading in Palmira - Valle

Plot	Genotype	Rep	Date	Plants fresh biomass (gr)	Plants Dry biomass (gr)	Hydric content (gr)	Panicle dry biomass (gr)	SPAD reading	Leaf temperature (ºC)
**8**	**G173**	1	12/17/2018	1481.54	237.08	84.00	241.29	40.84	31.08
**52**	**G97**	1	12/17/2018	1000.75	123.51	87.66	184.31	32.10	31.64
**65**	**G261**	1	12/17/2018	1185.52	164.12	86.16	192.47	34.86	32.22
**67**	**G52**	1	12/17/2018	1377.02	194.20	85.90	232.20	42.75	31.53
**84**	**G25**	1	12/17/2018	707.62	109.76	84.49	130.50	32.56	32.83
**158**	**G16**	1	12/17/2018	1195.06	193.04	83.85	241.01	29.72	26.58

**191**	**G185**	1	12/17/2018	1407.76	192.36	86.34	187.46	31.64	27.39
**208**	**G320**	1	12/17/2018	708.01	103.50	85.38	58.65	41.15	27.39
**254**	**G149**	1	12/17/2018	808.69	120.69	85.08	134.99	32.17	30.96
**268**	**G235**	1	12/17/2018	1077.45	160.99	85.06	212.87	25.65	30.60
**273**	**G204**	1	12/17/2018	623.90	109.05	82.52	100.57	28.28	28.83
**302**	**G58**	1	12/17/2018	662.06	112.91	82.95	117.21	37.75	30.19
**319**	**G71**	1	12/17/2018	988.06	152.03	84.61	168.93	40.50	31.22
**327**	**G61**	1	12/17/2018	1254.08	196.40	84.34	245.66	33.28	27.77
**385**	**G295**	1	12/17/2018	1250.12	213.04	82.96	253.48	33.43	28.05
**388**	**G29**	1	12/17/2018	883.06	141.99	83.92	134.09	36.23	30.44
**Mean**				**1038.17**	**157.79**	**84.70**	**177.23**	**34.56**	**29.92**

Table 21: Sampling 3 (12/21/2018) of dry biomass and SPAD reading in Palmira - Valle

Plot	Genotype	Rep	Date	Plants fresh biomass (gr)	Plants Dry biomass (gr)	Hydric content (gr)	Panicle dry biomass (gr)	SPAD reading	Leaf temperature (ºC)
**26**	**G302**	1	12/21/2018	1264.06	172.84	86.33	172.90	27.24	26.96
**32**	**G36**	1	12/21/2018	992.40	120.53	87.85	166.64	24.77	29.06
**70**	**G287**	1	12/21/2018	1369.02	213.48	84.41	165.23	28.03	27.58
**74**	**G289**	1	12/21/2018	1623.96	227.19	86.01	234.27	34.99	26.06
**83**	**G167**	1	12/21/2018	1495.20	204.55	86.32	238.17	31.53	27.64
**99**	**G174**	1	12/21/2018	1145.46	183.72	83.96	199.13	25.09	28.14
**106**	**G129**	1	12/21/2018	1096.56	149.25	86.39	192.92	32.98	25.69
**115**	**G41**	1	12/21/2018	1370.12	173.43	87.34	155.34	31.86	25.98
**134**	**G95**	1	12/21/2018	1483.19	193.99	86.92	228.06	18.97	27.82
**154**	**G90**	1	12/21/2018	1526.44	201.27	86.81	183.63	43.81	28.64
**161**	**G327**	1	12/21/2018	1181.80	135.98	88.49	132.90	45.20	27.89
**214**	**G155**	1	12/21/2018	1173.43	186.37	84.12	237.74	28.80	27.89
**232**	**G107**	1	12/21/2018	1402.76	178.72	87.26	186.55	30.67	26.40
**270**	**G122**	1	12/21/2018	1084.25	149.02	86.26	172.70	29.62	27.61
**286**	**G153**	1	12/21/2018	1168.07	177.72	84.79	164.76	31.29	26.44
**308**	**G125**	1	12/21/2018	993.40	159.70	83.92	143.83	28.86	28.80
**Mean**				**1273.13**	**176.74**	**86.07**	**185.92**	**30.86**	**27.41**
